# OligoFormer: an accurate and robust prediction method for siRNA design

**DOI:** 10.1093/bioinformatics/btae577

**Published:** 2024-09-25

**Authors:** Yilan Bai, Haochen Zhong, Taiwei Wang, Zhi John Lu

**Affiliations:** MOE Key Laboratory of Bioinformatics, Center for Synthetic and Systems Biology, School of Life Sciences, Tsinghua University, Beijing, 100084, China; Institute for Precision Medicine, Tsinghua University, Beijing, 100084, China; MOE Key Laboratory of Bioinformatics, Center for Synthetic and Systems Biology, School of Life Sciences, Tsinghua University, Beijing, 100084, China; Institute for Precision Medicine, Tsinghua University, Beijing, 100084, China; MOE Key Laboratory of Bioinformatics, Center for Synthetic and Systems Biology, School of Life Sciences, Tsinghua University, Beijing, 100084, China; Institute for Precision Medicine, Tsinghua University, Beijing, 100084, China; Academy for Advanced Interdisciplinary Studies (AAIS), and Peking University–Tsinghua University–National Institute of Biological Sciences Joint Graduate Program (PTN), Peking University, Beijing, 100871, China; MOE Key Laboratory of Bioinformatics, Center for Synthetic and Systems Biology, School of Life Sciences, Tsinghua University, Beijing, 100084, China; Institute for Precision Medicine, Tsinghua University, Beijing, 100084, China

## Abstract

**Motivation:**

RNA interference (RNAi) has become a widely used experimental approach for post-transcriptional regulation and is increasingly showing its potential as future targeted drugs. However, the prediction of highly efficient siRNAs (small interfering RNAs) is still hindered by dataset biases, the inadequacy of prediction methods, and the presence of off-target effects. To overcome these limitations, we propose an accurate and robust prediction method, OligoFormer, for siRNA design.

**Results:**

OligoFormer comprises three different modules including thermodynamic calculation, RNA-FM module, and Oligo encoder. Oligo encoder is the core module based on the transformer encoder. Taking siRNA and mRNA sequences as input, OligoFormer can obtain thermodynamic parameters, RNA-FM embedding, and Oligo embedding through these three modules, respectively. We carefully benchmarked OligoFormer against six comparable methods on siRNA efficacy datasets. OligoFormer outperforms all the other methods, with an average improvement of 9% in AUC, 6.6% in PRC, 9.8% in F1 score, and 5.1% in PCC compared to the best method among them in our inter-dataset validation. We also provide a comprehensive pipeline with prediction of siRNA efficacy and off-target effects using PITA score and TargetScan score. The ablation study shows RNA-FM module and thermodynamic parameters improved the performance and accelerated convergence of OligoFormer. The saliency maps by gradient backpropagation and base preference maps show certain base preferences in initial and terminal region of siRNAs.

**Availability and implementation:**

The source code of OligoFormer is freely available on GitHub at: https://github.com/lulab/OligoFormer. Docker image of OligoFormer is freely available on the docker hub at https://hub.docker.com/r/yilanbai/oligoformer.

## 1 Introduction

RNA interference (RNAi) makes small interfering RNAs (siRNAs) promising therapeutic drugs due to their potential to silence disease-related genes in a sequence-specific manner (Hu *et al.* 2020). siRNAs are introduced into the RNA-induced silencing complex (RISC), which comprises several distinct proteins, including Argonaute-2 (Ago-2), Dicer, and the transactivation response element RNA-binding protein (TRBP) (Zamore *et al.* 2000). After the activation of siRNAs through removal of their sense strands, the resulting antisense strands direct the RISC to bind with the target mRNA, where Ago-2 facilitates cleavage (Wilson and Doudna 2013). Upon cleaving the target mRNA, the siRNA-loaded RISC can dissociate and engage with another mRNA molecule. Consequently, minimal concentrations of siRNAs effectively induce gene knockdown. Notably, siRNAs exert their influence post-transcriptionally at the mRNA level, offering a distinct advantage over post-translational protein-focused approaches. This feature allows for the targeting of “undruggable genes” for which inhibitors are unavailable or challenging to develop, significantly expanding the range of potential therapeutic targets beyond traditionally druggable proteins (Friedrich and Aigner 2022). As of 2022, 10 RNAi drugs have been approved by the FDA or entered late-phase three clinical trials (Zhu *et al.* 2022), indicating the great potential of RNAi drugs in the future.

However, obtaining highly efficient siRNAs is a challenging task, requiring specific characteristics such as the absence of innate immune system activation, high efficacy in cutting specific targets, and minimal off-target or toxic effects (Friedrich and Aigner 2022). Hence, the design of effective siRNAs is crucial for the success of RNAi therapeutics, leading to the development of various computational tools to aid in this process. While many tools, such as OligoWalk (Lu and Mathews 2008c), siRNAPred (Kumar *et al.* 2009), DSIR (Vert *et al.* 2006), and i-score (Ichihara *et al.* 2007), have been tested and demonstrated effectiveness, they still exhibit some shortcomings. These can be broadly discussed in terms of dataset biases, prediction methods, and off-target effects.

Dataset biases may affect the generalization and validity of siRNA design models. Huesken *et al.* (2005) collected 2431 siRNAs targeting 34 mRNAs along with their inhibition values, making a significant contribution to the enrichment of siRNA datasets. LASSO-based regression model by Vert *et al.* (2006), DSIR, was trained primarily on this dataset. While they achieved rather good results, they lacked validation on other datasets, raising concerns about their generalization ability. Another important consideration is the significant variation in experimental conditions and data quality among different datasets. For example, Huesken *et al.* utilized a high-throughput fluorescent reporter gene system, while Hsieh *et al.* employed quantitative real-time PCR for measurement (Hsieh *et al.* 2004). Normalizing data across diverse datasets and integrating them appropriately pose significant challenges. The effectiveness of the final result is influenced by the characteristics of the prediction methods or models. Early researchers endeavored to employ machine learning methods for the analysis of siRNA efficacy. Huesken *et al.* used the Stuttgart Neural Net Simulator to train an algorithm named BIOPREDsi. Ichihara *et al.* developed a simple linear regression model, i-Score, to predict active siRNAs while considered only nucleotide preferences at each position. Lu *et al.* constructed a support vector machine (SVM) that selected functional siRNAs based on both thermodynamic and sequence features (Lu and Mathews 2008a,b). Recently, Monopoli *et al.* used asymmetric trichotomous partitioning to overcome dataset limitations based on random forest models for siRNA efficacy prediction (Monopoli *et al.* 2023). Since their final model was not named in the original paper, we called it Monopoli-RF for short here. It would be important to benchmark OligoFormer to these tools. While these methods achieved good results at the time, the structures of these models were relatively simple, and they could not extract some hidden features well. With the rise and proven capabilities of deep learning, researchers started applying deep learning models to biological problems, aiming to capture deep-dimensional features for analysis. Han *et al.* utilized convolution kernels as motif detectors to extract siRNA sequence features. After combining thermodynamic properties with a pooling layer, they introduced a deep neural network (DNN) to generate feature representations and output efficacy through a logistic regression function (Han *et al.* 2018). The performance of their proposed method was improved on the same dataset of Huesken *et al.* Apart from this, Massimo *et al.* proposed a graph neural networks (GNN) approach to simulate the siRNA-mRNA interaction networks. siRNAs and mRNAs were encoded with 3-mer and 4-mer fragments respectively as two types of nodes whereas 22 thermodynamic parameters calculated from RNAUp web server and Gibbs energy served as the third node (La Rosa *et al.* 2022). They took the prediction of siRNA efficacy to a new level. However, due to the limited complexity of these models, they still struggled to capture the binding properties between the long context of mRNAs and siRNAs. In recent years, transformer-based models have shown remarkable success in various natural language processing and genomics tasks compared to earlier deep learning models (Vaswani *et al.* 2017). siRNA sequences share similarities with sentences in language tasks, making transformers a probable choice. Furthermore, pretrained language models are starting to demonstrate their powerful capabilities in downstream migration tasks. RNA-FM is the first foundation model for the community to accommodate all non-coding RNA sequences (Chen 2022). These pretrained RNA models may enrich the feature representation of siRNA–mRNA interactions.

Off-target prediction is quite vital for the practical application of siRNAs in treatment. However, many existing prediction models only focus on the effectiveness of binding predicted siRNAs to target mRNA to produce inhibitory effects, overlooking the potential off-target effects caused by the siRNA sequence itself. The off-target effects associated with siRNAs delivery can be divided into three broad categories: miRNA-like off-target, immune stimulation, and saturation of the RNAi mechanism (Jackson and Linsley 2010). siRNAs, with seed region complementarity acting like miRNAs, may down-regulate a large number of transcripts (Jackson *et al.* 2006). While chemical modification of the seed region can reduce the effects (Jackson *et al.* 2006), a comprehensive transcriptome comparison remains necessary. For predicting the miRNA-like off-target effects, well-established miRNA-target prediction tools can be applied in this field (Riba *et al.* 2017). Immune stimulation mainly results from specific motifs in the siRNA strand, such as UGUGU, GUCCUUCA, and CUGAAUU (Fakhr *et al.* 2016). Regarding RNAi mechanism saturation, no strategies have been identified to mitigate this effect.

Here, we propose a novel method for predicting siRNA efficacy and off-target effects, named OligoFormer. This method consists of two components: a transformer-based model to capture deep hidden sequence features and learn complex patterns of siRNA–mRNA interactions for siRNA efficacy prediction, and an overall pipeline to select the best siRNA predicted by our model, taking into account various off-target effects.

## 2 Materials and methods

### 2.1 Dataset collection and preprocessing

#### 2.1.1 Dataset collection

We strategically aggregated a diverse array of datasets to facilitate accuracy and robustness of our model. We collected nine datasets comprising a total of 3714 siRNAs and 75 mRNAs from previous studies, including those by Huesken *et al.* (2005), Katoh and Suzuki (2007), Amarzguioui *et al.* (2003), Harborth *et al.* (2003), Hsieh *et al.* (2004), Reynolds *et al.* (2004), Vickers *et al.* (2003), and Ui-Tei *et al.* (2004) (Table 1). We categorized the datasets into three sets: the Huesken dataset, the Takayuki dataset, and the merged remaining datasets as Mixset (Supplementary Table S1.1). The inhibition efficacy or activity of siRNAs in all datasets was normalized into inhibition efficiency ranging from 0% to 100%, and 70% of the original maximum inhibition was used as the threshold to classify positive and negative siRNAs. We employed Needleman–Wunsch global alignment algorithm (Needleman and Wunsch 1970) to remove one redundant RNA sequence in Mixset with more than 80% identity between the training and test sets.

**Table 1.** The datasets of siRNAs.

Sources	siRNA^a^	mRNA^a^	Cell line^b^	Datasets
Huesken	2431	34	H1299	Huesken
Takayuki	702	1	HeLa	Takayuki
Amarzguioui	46	4	HaCaT	Mixset
Harborth	44	1	HeLa	
Hsieh	108	22	HEK293T	
Khvorova	14	1	HEK293	
Reynolds	240	7	HEK293	
Vickers	76	2	T24	
Ui-Tei	53	3	HeLa	

a This represents the number of siRNAs or mRNAs.

b Only the most representative cell line was selected.

#### 2.1.2 Flanking region of target mRNAs

We selected the complementary region of the 19 nucleotides (nt) siRNA as the central point and extended it in both directions to obtain the corresponding mRNA sequences. The selection of an appropriate flanking region surrounding the target mRNA sequence is important for efficient and specific mRNA embeddings. We systematically investigated the impact of flanking region length on siRNA efficacy prediction. The 5′ and 3′ extension regions traverse from 1 to 100 nt long, respectively, and the performance of each combination for siRNA efficacy prediction was evaluated. For symmetric flanking regions, we observed that flanking length of 19 nt outperformed the other symmetric flanking lengths and the model’s performance fluctuated within a small range around 19 nt. For asymmetric flanking regions, the model stably performed well when both flanking regions were approximately 15–24 nt long (Supplementary Fig. S4 and Table S2). There are two possible reasons. Firstly, longer flanking regions provide more context of mRNA but dilute the specific information of siRNA sequences, and *vice versa*. The 19 nt flanking region may strike a balance between providing sufficient context of mRNA sequences and minimizing dilution of siRNA information. Secondly, the flanking length of 19 nt aligns with the processing length of siRNA, which facilitates the feature extraction of the BiLSTM (bidirectional long short-term memory) layer. Therefore, 19 nt was chosen as the length of the flanking region of the mRNAs.

#### 2.1.3 Sequence normalization

To facilitate model training, it is essential to ensure consistent lengths for both siRNAs and mRNAs across different datasets. Specifically, we uniformly truncated siRNA sequences to 19 nucleotides. In most datasets, siRNAs were naturally 19 nucleotides long, while in the Huesken dataset, they were 21 nucleotides long and the two nucleotides at positions 20–21 in the 3′-overhang were truncated to ensure uniformity. For mRNA sequences, extensions of upstream and downstream parts less than 19 nucleotides are padded with the X nucleotide. This normalization method enables consistent processing and feature extraction across all datasets (Supplementary Fig. S6).

#### 2.1.4 RNA-FM model embedding

In this study, we utilized RNA language model RNA-FM to generate embeddings for both siRNAs and mRNAs, serving as additional features. RNA-FM can extract various structural and compositional features of RNA, capturing nucleotide information from both primary sequence and secondary structure. The embeddings generated by RNA-FM were used as additional high-dimensional input features for OligoFormer, enhancing the model’s ability to learn relationships between siRNAs and mRNAs. The shape of the embedding is determined by the length of the RNA sequences multiplied by 640. Therefore, the shape of the siRNA embedding is 19 × 640, and the shape of the mRNA embedding is 57 × 640. All RNAs in our datasets were embedded and pre-saved in the npy format using RNA-FM for subsequent model training.

#### 2.1.5 Thermodynamic parameters

**Figure 1.**
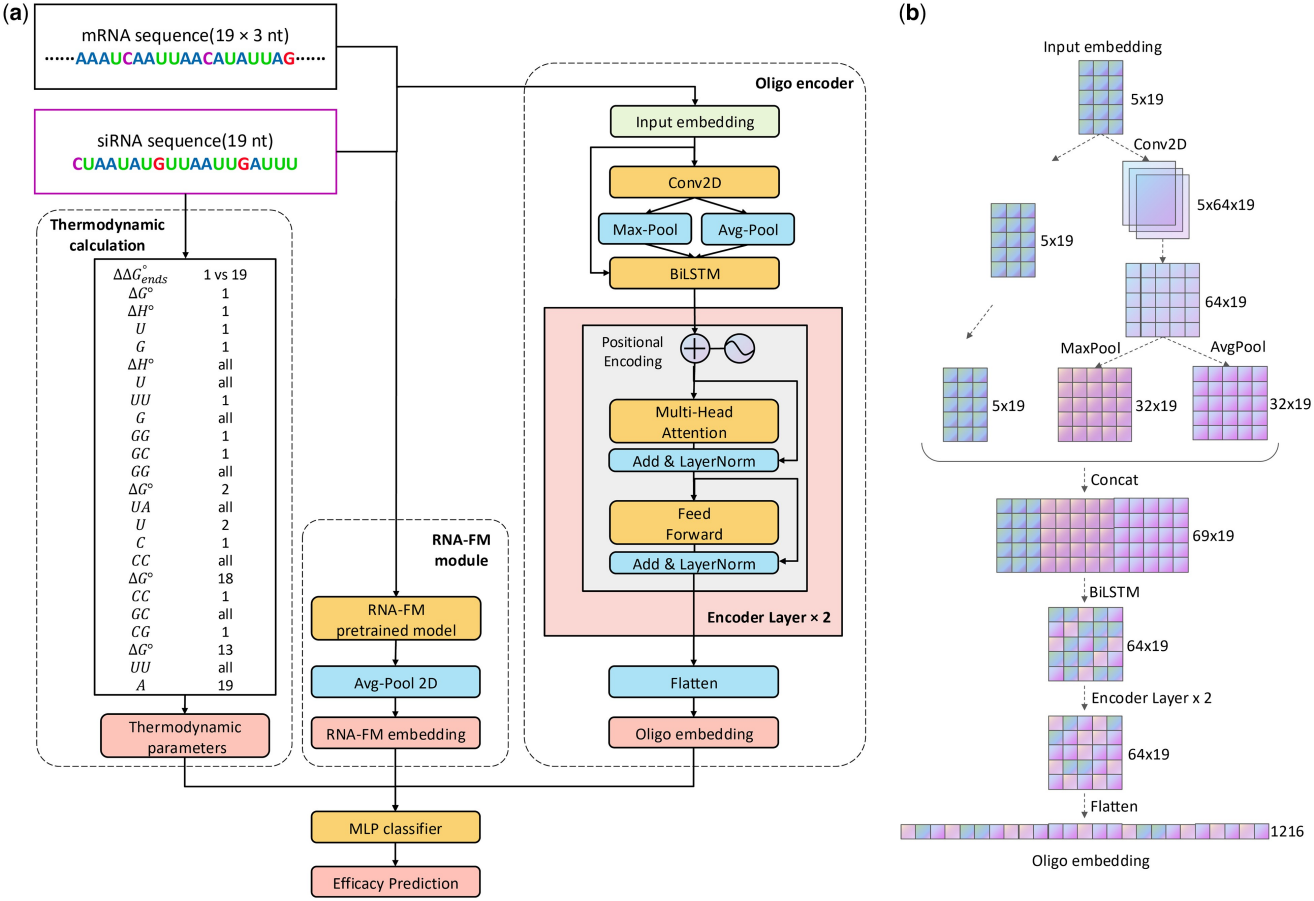
Overview of OligoFormer. (a) The architecture of OligoFormer. All features including thermodynamic parameters, RNA-FM embedding, and Oligo embedding, are first calculated from siRNA and mRNA sequences through different modules and then concatenated together into MLP classifier to obtain the prediction of siRNAs. The Oligo encoder comprises a 2D convolution layer, a max pooing layer, an average pooling layer, a bidirectional LSTM, two-layer multi-head transformer encoder, and a flatten layer to get Oligo embedding. (b) The computational graph illustrates the detailed structure of Oligo encoder and the shape of RNA embedding at each layer of Oligo encoder for siRNA, using 64 as the example dimension of the hidden layer.

In addition to standard sequence embeddings, OligoFormer integrates RNA thermodynamic parameters as crucial input features. These features (Fig. 1a; Supplementary Table S1.2) were computed for each siRNA sequence, providing valuable insights into the thermodynamic stability and binding affinity of siRNA–mRNA interactions (Supplementary Methods S1.2 ). The nucleotide content provides information about the stability of the interaction, indicating the conditions under which the siRNA effectively binds to the mRNA. Furthermore, Δ*G* quantifies the free energy change associated with the formation of the siRNA itself and the duplex, providing a measure of the thermodynamic favorability of the binding (Supplementary Table S1.4). Lower values of Δ*G* suggest more energetically favorable interactions. These thermodynamic parameters represent some intrinsic properties of RNA and provide a comprehensive foundation for siRNA efficacy prediction. Integrating them into the model’s input features allows OligoFormer to gain a better understanding of the energetics governing siRNA functionality.

### 2.2 The architecture of OligoFormer

OligoFormer takes siRNA and mRNA sequences as input and comprises three different modules including thermodynamic calculation, RNA-FM module, and Oligo encoder (Fig. 1a). Thermodynamic parameters, RNA-FM embedding, and Oligo embedding will be calculated for siRNA and mRNA, respectively, from individual processing through these modules, and OligoFormer employs a late fusion mechanism to combine diverse features by concatenated into a multilayer perceptron (MLP) classifier with equal weights. This architecture allows OligoFormer to capture both individual features and their combined impact on siRNA efficacy. A pivotal module of OligoFormer is Oligo encoder, which is responsible for distilling complex information from the input features and generating a comprehensive Oligo embedding. The detailed structure of the Oligo encoder consists of a sequence of layers tailored for different aspects of feature extraction (Supplementary Table S1.3). Starting with a 2D convolutional layer, the encoder captures spatial dependencies within the input features, which is followed by a max pooling layer and an average pooling layer, extracting features while retaining the most relevant information. The BiLSTM layer introduces temporal dynamics and captures sequential dependencies. A two-layer multi-head transformer encoder further enhances the model’s ability to capture complex patterns by leveraging attention mechanisms. Lastly, a flatten layer consolidates the extracted features to obtain a comprehensive RNA embedding. The siRNA and mRNA have their own independent Oligo encoder. Their parameters are independent and not shared with each other, allowing each encoder to capture the unique features of siRNA and mRNA sequences. To visually illustrate the embedding flow, we present a computational graph detailing the shape of the RNA embedding at each layer of the Oligo encoder for siRNA (Fig. 1b). With a dimension of 64 for the hidden layers, the graph illustrates how the features change from input embedding of siRNA to Oligo embedding.

### 2.3 Cross-validation methods

We compared OligoFormer with previous open source methods, including Monopoli-RF, OligoWalk, siRNAPred, i-score, s-Biopredsi, and DSIR on both intra-dataset and inter-dataset validation (Supplementary Table S3). Other softwares of commercial companies, such as siRNA wizard (https://www.invivogen.com/sirnawizard/), siDesign Center (Nagapoosanam *et al.* 2019) and siRNA Target Finder (Wang and Mu 2004) were not compared here because they are not open source.

For intra-dataset validation, we employed a 5-fold cross-validation strategy on the Huesken, Mixset, and Takayuki datasets to mitigate biases and ensure robustness. Each dataset was split into five subsets, with each subset serving as a validation set exactly once to evaluate models’ performance across diverse data splits. OligoFormer and the other six methods were employed using this validation scheme to assess their performance on individual datasets, which provided a basic standard to measure the accuracy and robustness of these models.

For inter-dataset validation, we employed it to assess the model’s generalization and robustness across different datasets. We trained models on the Huesken dataset and evaluated their performance on the Mixset dataset. We also performed cross cell line validation to ensure that our model could maintain robust performance when applied to different cell lines (Supplementary Table S1.5). The ability of models to provide accurate predictions across diverse datasets is crucial for their applicability in real-world scenarios with varying experimental conditions.

## 3 Results

### 3.1 Performance on intra-dataset validation

**Figure 2.**
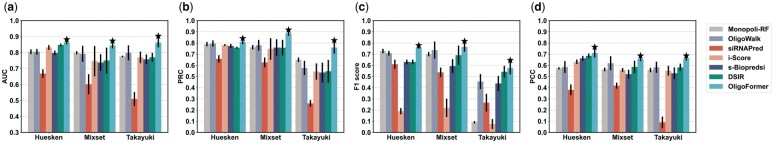
The performance on intra-dataset validation. (a) The AUC values of OligoFormer and the other six methods on Huesken, Mixset, and Takayuki datasets in 5-fold validation. The black vertical line represents standard error bar, and the star above it indicates the best AUC. (b) The PRC values of OligoFormer and the other six methods on Huesken, Mixset, and Takayuki datasets in 5-fold validation. (c) The *F*1 scores of OligoFormer and the other six methods on Huesken, Mixset, and Takayuki datasets in 5-fold validation. (d) The PCC of OligoFormer and the other six methods on Huesken, Mixset, and Takayuki datasets in 5-fold validation.

We compared OligoFormer with other siRNA design models, including Monopoli-RF, OligoWalk, siRNAPred, i-score, s-Biopredsi, and DSIR. The performance of these models was evaluated on three datasets, Huesken, Mixset, and Takayuki datasets, using 5-fold cross-validation. AUC (area under curve), PRC (area under the precision-recall curve), *F*1 score, and PCC (Pearson correlation coefficient) were used as metrics to measure model performance. OligoFormer outperforms other methods on all three datasets (Fig. 2 and Supplementary Fig. S2), achieving an average AUC of 0.8619, an average PRC of 0.8099, an average *F*1 score of 0.7584, and an average PCC of 0.7114 on Huesken dataset, an average AUC of 0.8454, an average PRC of 0.8858, an average *F*1 score of 0.7641, and an average PCC of 0.6564 on Mixset, and an average AUC of 0.8628, an average PRC of 0.7586, an average *F*1 score of 0.577, and an average PCC of 0.6596 on Takayuki dataset (Table 2). OligoFormer stands out among other methods on intra-dataset validation, demonstrating its accuracy in learning and understanding one certain dataset.

**Table 2.** Performance comparison among different siRNA design data methods for intra-dataset and inter-dataset validation.

Methods	Huesken dataset^a^				Mixset				Takayuki dataset				Inter-dataset			
	AUC	PRC	F1 score	PCC	AUC	PRC	F1 score	PCC	AUC	PRC	F1 score	PCC	AUC	PRC	F1 score	PCC
Monopoli-RF^b^	0.805	0.7893	**0.7276**	0.5731	0.7984	0.7621	0.7001	0.5633	0.7756	**0.6498**	0.0909	0.5578	0.7439	0.7388	**0.7002**	0.4628
OligoWalk	0.8034	**0.795**	0.7083	0.5847	**0.7919**	**0.7793**	**0.7364**	**0.6191**	**0.7979**	0.5764	0.4548	**0.5829**	0.708	0.6998	0.6258	**0.5592**
siRNAPred	0.6681	0.658	0.6097	0.3809	0.6014	0.6257	0.5371	0.4179	0.5094	0.2595	0.2662	0.09	0.5782	0.618	0.5245	0.2062
iScore	0.8314	0.7813	0.1896	0.6318	0.7462	0.7465	0.2197	0.5581	0.7695	0.5443	0.07572	0.5517	0.7462	0.7565	0.2197	0.3687
s-Biopredsi	0.7975	0.7731	0.6299	0.6621	0.7374	0.7589	0.5936	0.5207	0.7576	0.5358	0.4379	0.5287	0.7374	0.7557	0.5936	0.3849
DSIR	**0.8472** ^c^	0.7575	0.6299	**0.6846**	0.7498	0.7575	0.6905	0.5862	0.7702	0.5469	0.5422	0.5815	**0.7483**	**0.7637**	0.6945	0.503
OligoFormer	**0.8619**	**0.8099**	**0.7584**	**0.7114**	**0.8454**	**0.8858**	**0.7641**	**0.6564**	**0.8628**	**0.7586**	**0.5769**	**0.6596**	**0.8163**	**0.8143**	**0.769**	**0.5879**

a The first nine columns represent intra-dataset training and each value is the mean of 5-fold cross-validation.

b Using two partition method for Monopoli-RF training.

c Bold text represents the top two methods based on the evaluation metrics.

### 3.2 Performance on inter-dataset validation

In addition to the ability to understand one certain dataset, the model’s predictive performance across datasets is even more crucial. In our inter-dataset comparison, OligoFormer was trained on the Huesken dataset and validated on the Mixset and Takayuki datasets, alongside OligoWalk, siRNAPred, i-score, s-Biopredsi, and DSIR. AUC and *F*1 score were used to evaluate the performance of each model across different datasets. OligoFormer outperforms the other models on all valid datasets (Supplementary Fig. S2), achieving an average AUC of 0.8163, an average PRC of 0.8143, an average *F*1 score of 0.769, and an average PCC of 0.5879 on the Mixset dataset (Table 2). Therefore, OligoFormer is more robust for varying sequence characteristics, experimental conditions, and assay methodologies than other methods.

### 3.3 Ablation study

**Figure 3.**
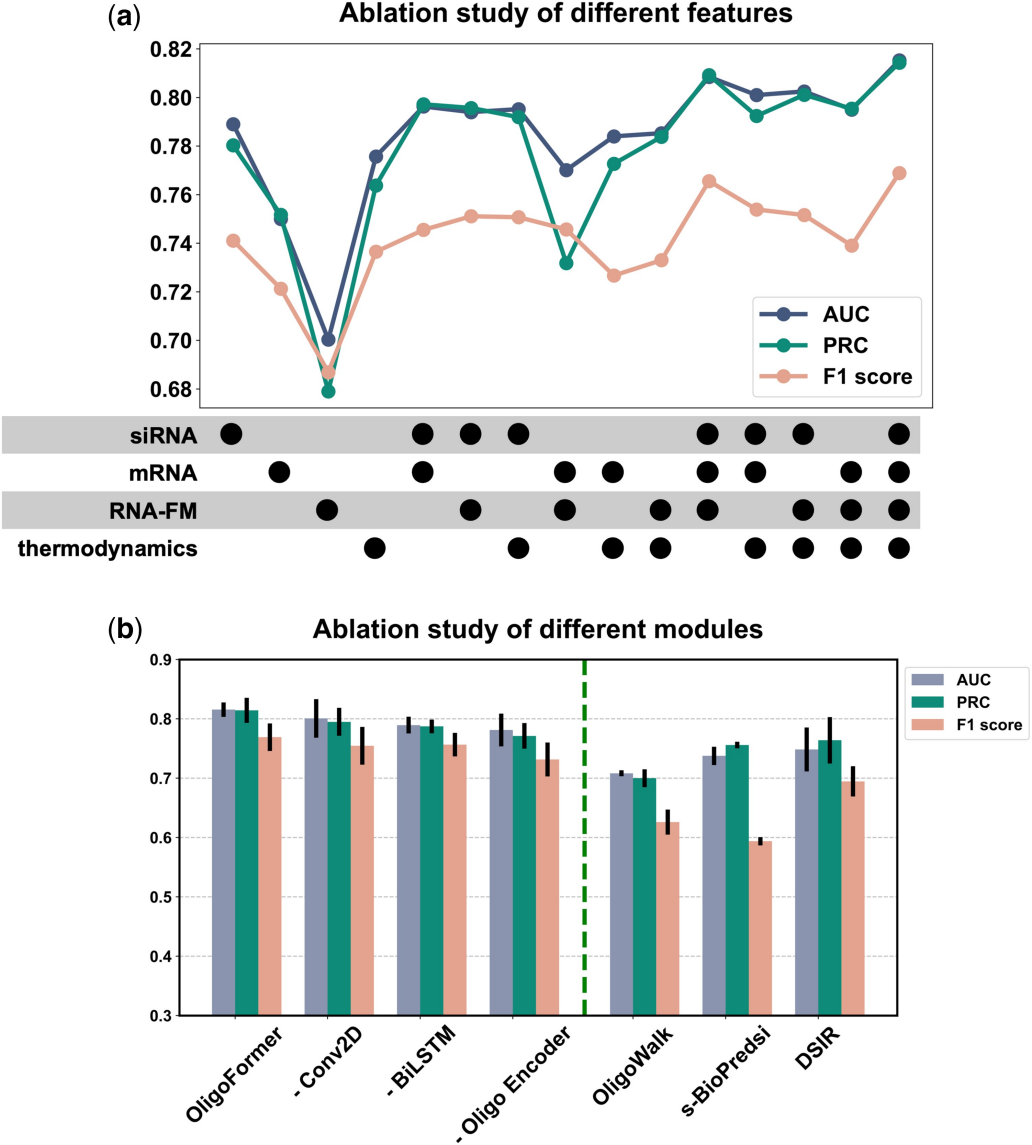
Ablation analysis of different input features and modules of OligoFormer. (a) Ablation study of different feature combinations of OligoFormer. Points represent the addition of corresponding features. (b) Ablation study of different modules of OligoFormer. The left side of the dotted line is the complete OligoFormer and uncomplete OligoFormer with the corresponding module removed. The right side of the dotted line is previous methods as benchmarks.

Ablation study was conducted to evaluate the impact of different features and modules on the performance of OligoFormer based on inter-dataset validation using AUC, PRC, and *F*1 score. We examined the effects of different combinations of four types of features: siRNA sequences, mRNA sequences, RNA-FM embeddings, and thermodynamic parameters (TD). Fifteen feature sets in total were generated by combining these feature types. The results indicated that the combination of all input features achieved the best performance (Fig. 3a). Removing any module would cause the model to become less accurate, and a notable decline in performance was observed when RNA-FM and siRNA features were removed, which means these two features are crucial for capturing the complexities of RNA interactions. The incorporation of TD enriched the features used for prediction and allowed OligoFormer to capture a broader spectrum of information, enabling a more accurate evaluation of siRNA efficacy. The mRNA sequence was another important feature that provides context for siRNA binding. All these features contributed to the superior performance of OligoFormer.

Then, we evaluated the essentialities of different modules in OligoFormer (Supplementary Table S5). Here, we present part of the results including the complete OligoFormer, the model with Conv2D removed, the model with BiLSTM removed, and Oligo encoder removed model with previous methods as benchmarks. The complete OligoFormer model achieved the best performance, followed by -Conv2D, -BiLSTM, and -Oligo encoder, successively (Fig. 3b). The model’s performance decreased the most when the Oligo encoder was removed, highlighting its indispensability. The Oligo encoder uses a transformer encoder to capture intricate patterns and dependencies in sequential data, making it well-suited for the complex nature of siRNA sequences. We also measured how these different features influence the convergency speed of our models. Adding mRNA features slowed down the convergence speed, and incorporating RNA-FM and thermodynamic parameters accelerated the model’s convergence (Supplementary Fig. S1). The addition of mRNAs appears to introduce complexities that hinder the optimization process, leading to a slower convergence speed. This could be due to the introduction of noise that disrupts the learning dynamics. RNA-FM and thermodynamic parameters likely contribute positively to the convergence speed. As a foundation model, RNA-FM enhances the model’s ability to capture features of RNA sequences and provide valuable representations. And thermodynamic parameters may enable the model to exploit energy-based considerations, guiding the optimization process more efficiently.

### 3.4 Model interpretation

**Figure 4.**
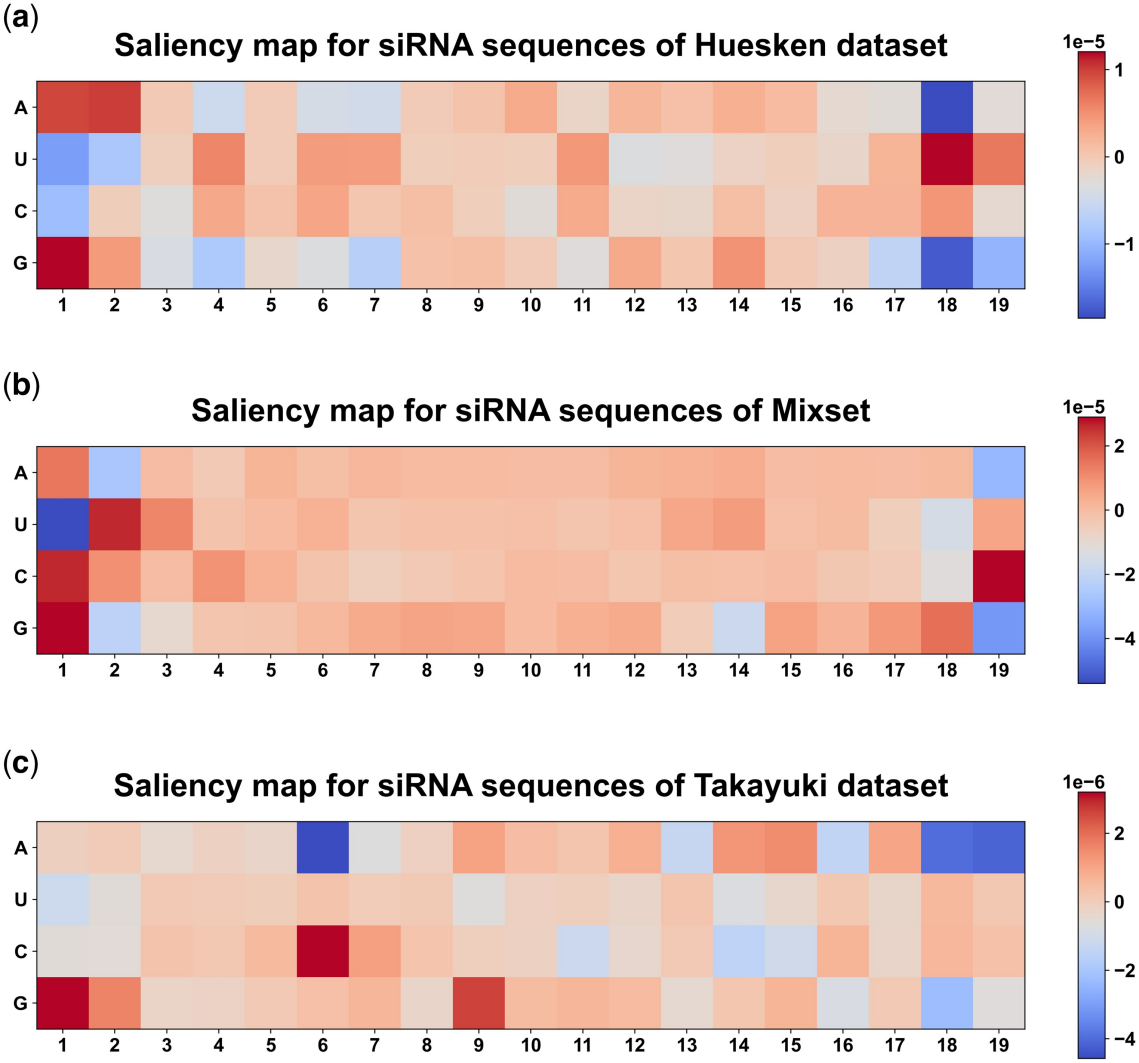
Saliency map for siRNAs on (a) Huesken, (b) Mixset, (c) Takayuki dataset. Larger numbers represent higher gradients and importance in the corresponding position.

Understanding the inner workings of models is crucial for gaining insights into the decision-making process. We visualized the interpretability of OligoFormer by generating the saliency map through gradient backpropagation on Huesken, Mixset, Takayuki datasets. Saliency map serves as a valuable tool to elucidate which parts of the input siRNAs significantly influence predictions of OligoFormer and trace the impact of each nucleotide feature back to the model’s input. Regions with higher saliency values indicate molecular components that play a substantial role in the model’s decision-making process. A and G bases in the first two positions of siRNAs showed high saliency value, while the U base in the last two positions of siRNAs showed high saliency value (Fig. 4). This phenomenon has been reported by previous researches (Ladunga 2007), which indicated that A and G in 1–2 position of siRNAs and U in 18–19 position of siRNAs showed a high PCC with the efficacy. We also plotted base preference maps of OligoFormer to show the frequency and importance of specific nucleotides at each position (Supplementary Fig. S5).

### 3.5 OligoFormer with off-target searching for siRNA design

**Figure 5.**
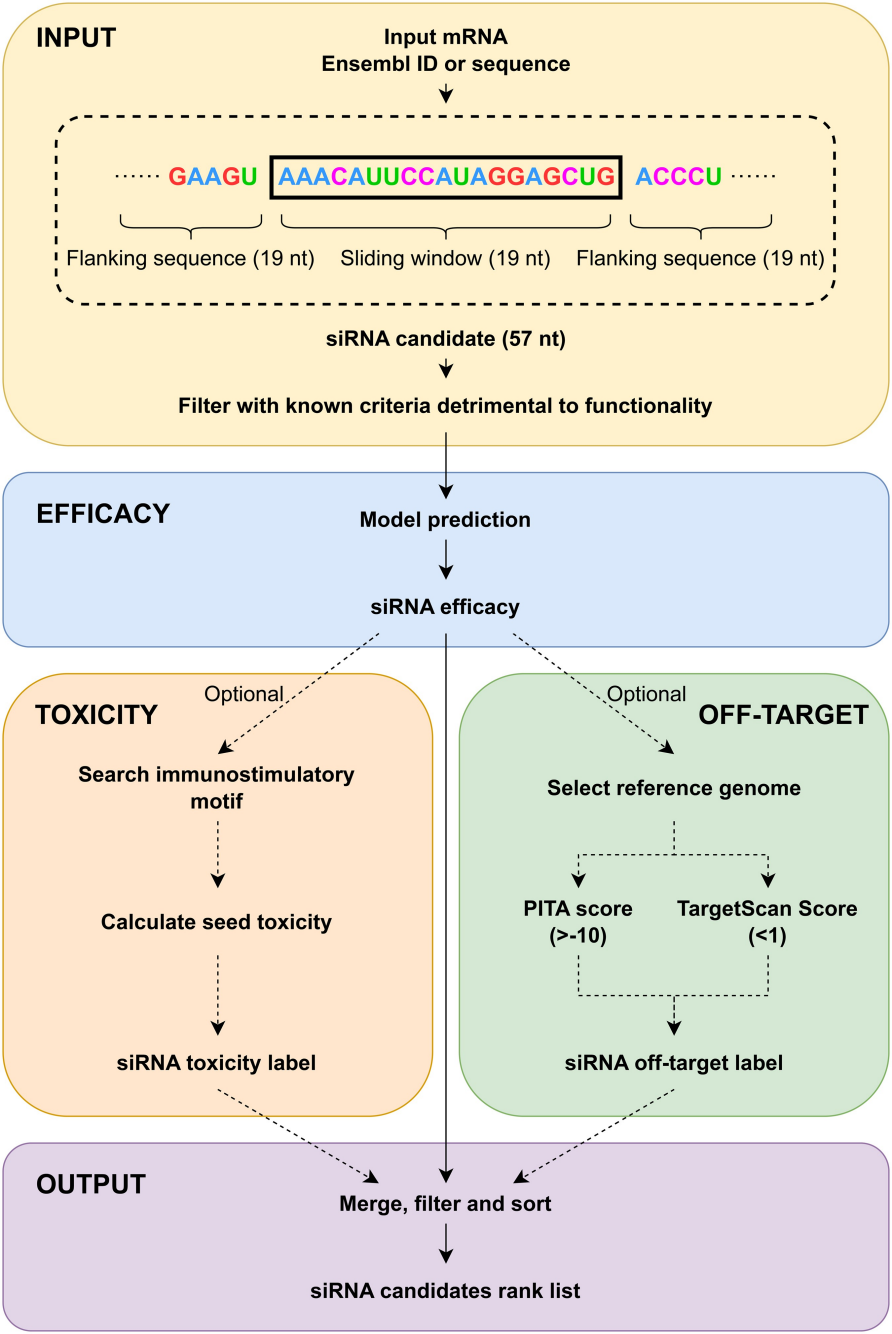
The pipeline of OligoFormer with off-target searching for siRNA design. Given a target mRNA as the input, siRNA efficacy, toxicity label, and off-target label will be calculated and merged to get a ranked list of siRNA candidates.

We also incorporated two existing off-target tools into OligoFormer to achieve better siRNA design (Fig. 5). This pipeline combines OligoFormer with off-target searching to provide an accurate prediction of siRNA efficacy and off-target effects.

OligoFormer can efficiently infer effective siRNAs given a target mRNA as the input. During the inference process, a sliding window of 19 nucleotides will be utilized to scan the whole target mRNA sequence and generate a set of potential siRNA candidates. To ensure the activity of the siRNAs, some criteria known to be detrimental to RNAi functionality will be applied first to filter the candidates (Supplementary Methods S1.9). Later each siRNA candidate is expanded to its corresponding 57-nucleotide mRNA sequence and the corresponding thermodynamic parameters of these siRNA candidates will be calculated. Then OligoFormer incorporates RNA-FM embedding vectors as pretrained representations of siRNA sequences. Next, OligoFormer will feed these diverse features into the pretrained OligoFormer neural network to predict the efficacy of siRNA candidates. The output of the model represents a prediction score for each siRNA, reflecting its potential effectiveness in inducing gene knockdown.

The off-target effects and toxicity associated with each siRNA are also incorporated into the pipeline to ensure a comprehensive assessment. Any siRNA candidate with motifs which specifically induce immune response is prioritized for filtration. siRNAs with the toxic seed sequence are subsequently eliminated (Supplementary Methods S1.9). These sequences to be filtered will receive a toxicity label to facilitate subsequent screening. Then some existing miRNA-target prediction methods can be applied to evaluate miRNA-like off-target potential of siRNAs. TargetScan Context++ (Agarwal *et al.* 2015) provides a quantitative model incorporating 14 features for miRNA targeting efficacy prediction, as well as miRNA-like off-target effects, which is the primary module of siRNA off-target effects. PITA (Kertesz *et al.* 2007) is a parameter-free model for miRNA-target interaction prediction based on site accessibility. It considers the difference between the gained free energy from the miRNA-target formation and the energetic cost of opening up the original base pairings of target RNA. Moreover, PITA is a reliable method for siRNA off-target prediction (Riba *et al.* 2017). For each filtered siRNA candidates with a predicted efficacy provided by OligoFormer, two off-target scores are calculated by TargetScan Context++ and PITA, respectively, within a given interested mRNA set, serving as the basis for customized filtration. Sequences that exceed the off-target threshold will receive an off-target label. We also utilize the existing siRNA off-target microarray data to verify the effectiveness of the combination of these two off-target prediction methods (Supplementary Methods S1.9 and Fig. S3).

The final output is a ranked list of siRNAs sorted by the predicted efficacy with filtering based on off-target markers and toxicity markers.

## 4 Discussion

Compared with previous methods, OligoFormer comprehensively considers RNA sequence features by the Oligo encoder, pretrained features by RNA-FM, and thermodynamic parameters. We believe there are three main reasons why our model outperforms the others on both intra-dataset and inter-dataset validation. Firstly, to the best of our ability, we collected and normalized all published siRNA datasets to account for different dataset characteristics, ensuring a robust and diverse training set. Secondly, we successfully applied the transformer model to predict siRNA efficacy through the Oligo encoder, which leverages the power of transformers to enhance the efficiency and accuracy of siRNA design in various applications. According to our ablation study, the Oligo encoder contributed most to the model’s performance. Thirdly, we utilized RNA-FM to generate pretrained RNA representations, which accelerated convergence speed and improved model performance. Additionally, we considered off-target siRNAs and developed a comprehensive pipeline for analyzing off-target effects in siRNA design.

However, there is still plenty of room for improvement. The limited representation and quantity of existing datasets constrain the effectiveness and generalization of siRNA design models. Therefore, it is necessary to make more datasets public or build new datasets. Currently, other pretrained RNA language models are gaining popularity. For instance, RNA-MSM is an unsupervised RNA language model based on multiple sequences that outputs both embeddings and attention maps (Zhang *et al.* 2024). Given that siRNAs and mRNAs do not interact in the same way as non-coding RNAs, other models may provide better characterization of this problem. Chemical modification is another important strategy to reduce the off-target effect of siRNAs, but it has not yet been included in the prediction process at present. If chemical modification predictions can be encoded and decoded using published data, it may significantly improve the prediction of siRNA efficacy and off-target effects, thereby facilitating its application in siRNA design.

This research provides an accurate and robust transformer-based method for siRNA design. We hold the belief that OligoFormer will provide scientific and comprehensive advice for researchers and help their oligo formed.

## Supplementary Material

btae577_Supplementary_Data

## Data Availability

All datasets used in this study are publicly available for academic research usages. The details of methods are fully illustrated in Supplementary Materials. Source code of OligoFormer is freely available on Github with detailed instructions at https://github.com/lulab/OligoFormer. Docker image of OligoFormer can be accessed on the docker hub at https://hub.docker.com/r/yilanbai/oligoformer.
