# Supplementary material for: OligoFormer: an accurate and robust prediction method for siRNA design
Source: Bioinformatics. 2024 Sep 25;40(10):btae577. doi: 10.1093/bioinformatics/btae577 (PMC11494384; doi:10.1093/bioinformatics/btae577)
Supplement: btae577_Supplementary_Data [file btae577_supplementary_data.zip › Supplementary Materials.docx]

**﻿OligoFormer: an accurate and robust prediction method for siRNA design**

Yilan Bai^1,2^, Haochen Zhong^1,2^, Taiwei Wang^1,2,3^, Zhi John Lu^1,2,^ ^*^

^1^MOE Key Laboratory of Bioinformatics, Center for Synthetic and Systems Biology, School of Life Sciences, Tsinghua University, Beijing, China.

^2^Institute for Precision Medicine, Tsinghua University, Beijing, China.

^3^Academy for Advanced Interdisciplinary Studies (AAIS), and Peking University–Tsinghua University–National Institute of Biological Sciences Joint Graduate Program (PTN), Peking University, Beijing, China

*Corresponding author: Tel.: +86 10 62789217, E-mail: [zhilu@tsinghua.edu.cn](mailto:zhilu@tsinghua.edu.cn)

# Supplementary Methods

## Data preprocessing

### *Redundancy Removal*

### Redundancy removal is a crucial step in constructing training and test sets to avoid the data leakage. We employed Needleman-Wunsch global alignment algorithm (Needleman and Wunsch 1970) to remove redundant RNA sequences between the training and test sets. The Needleman-Wunsch algorithm is a dynamic programming method widely used for sequence alignment, and allows for the optimal alignment of two siRNAs by maximizing the identity between them while considering possible gaps and mismatches. We calculated the identity percentage of each siRNA or mRNA sequence in the test set with every siRNA or mRNA sequence in the training set.﻿ We removed 1 siRNA from the Mixset that had more than 80% identity with any sequence in the Huesken set. Detailed results of redunancy are available on our Github library.

### *Efficacy normalization*

### The efficacy of Huesken, Mixset, and Takayuki datasets range from 0 to 1.341, -0.278 to 0.99, and 0 to 0.98, respectively. Min-max scaler was utilized to normalize the efficacy into a standardized inhibition efficiency scale ranging from 0 to 100%, which ensures that siRNA efficacy values were standardized as regression machine learning labels and comparable across datasets. We set the threshold at 70% inhibition efficiency for siRNAs in the dataset to classify siRNAs as positive or negative based on their inhibition efficiency.

### *﻿Flanking region of target mRNAs*

The selection of an appropriate flanking region surrounding the target mRNA sequence is important for efficient and specific mRNA embeddings. We systematically investigated the impact of flanking region length on siRNA efficacy prediction. We selected the complementary region of the 19 nt siRNA as the central point and extended it in both directions to obtain the corresponding mRNA sequences. This extension was conducted iteratively, ranging from 1 nt to 100 nt. Two hyperparameters “lm1” and “lm2” were employed to represent for the 5’ and 3’ flanking length to adapt to the different processing dimensions of the BiLSTM. We evaluated the siRNA efficacy prediction across different lengths of flanking region, and observed that flanking length of 19 nt outperformed the other symmetric flanking lengths, and in the analysis of asymmetric flanking length, the model stably performed well when both flanking regions were approximately 15-24 nt long (Supplementary Fig. 4). There are two possible reasons. Firstly, longer flanking regions provide more context of target mRNA but dilute the specific information of siRNA sequences, and vice versa. 19 nt flanking region may strike a balance between providing sufficient context of mRNA sequences and minimizing dilution of siRNA information. Secondly, the flanking length of 19 nt aligns with the processing length of siRNA, which facilitates the feature extraction of the BiLSTM. Therefore, 19 nt was chosen as the length of the flanking region of the mRNAs for the downstream analysis.

## Thermodynamic parameters

The calculation of thermodynamic parameters is divided into two parts: frequency of single nucleotide or dinucleotide, and Gibbs free energy changes and enthalpy changes at specific position or over all siRNA. OligoFormer employs established algorithms to calculate thermodynamic parameters (Xia, SantaLucia et al. 1998, Ladunga 2007), including nearest-neighbor thermodynamic parameters, the Gibbs free energy (∆G), and enthalpy (∆H) of RNA duplex formation. The calculation equations for each thermodynamic parameter are described in detail below, and an example siRNA is shown for a better understanding of the calculation process.

### *Single nucleotide or dinucleotide*

The single nucleotide N with the number k is whether the nucleotide at position k of siRNA is N, and the dinucleotide NM with the number k is whether the nucleotides at position k and position k+1 of siRNA are NM. Single nucleotide N with “all” is the frequency of nucleotide N in siRNA, and dinucleotide NM with “all” is the frequency of nucleotide NM in siRNA.

$$\boldsymbol{N}\left( k \right)=\left\{ \begin{aligned} 0, &siRNA[k]\neq\boldsymbol{N} \\ 1, &siRNA[k]=\boldsymbol{N} \end{aligned} \right., \boldsymbol{N}\in\left[ \boldsymbol{A},\boldsymbol{U},\boldsymbol{C},\boldsymbol{G} \right], k\in\left[ 1,19 \right]$$

$$\boldsymbol{NM}(k)=\left\{ \begin{aligned} 0, &siRNA[k:k+1]\neq\boldsymbol{NM} \\ 1, &siRNA[k:k+1]=\boldsymbol{NM} \end{aligned}, \boldsymbol{N},\boldsymbol{M}\in\left[ \boldsymbol{A},\boldsymbol{U},\boldsymbol{C},\boldsymbol{G} \right],k\in[1,19] \right.$$

$$\boldsymbol{N}\left( all \right)=\frac{sum\left( \boldsymbol{N} \right)}{19}, \boldsymbol{N}\in\left[ \boldsymbol{A},\boldsymbol{U},\boldsymbol{C},\boldsymbol{G} \right]$$

$$\boldsymbol{NM}\left( all \right)=\frac{sum\left( \boldsymbol{NM} \right)}{18}, \boldsymbol{N},\boldsymbol{M}\in\left[ \boldsymbol{A},\boldsymbol{U},\boldsymbol{C},\boldsymbol{G} \right]$$

Take the first siRNA in Huesken dataset “CUAAUAUGUUAAUUGAUUU”, for example,

$$\mathbf{U}\left( 1 \right)= 0, \mathbf{G}\left( 1 \right)= 0, \mathbf{UU}\left( 1 \right)= 0, \mathbf{GG}\left( 1 \right)=0, \mathbf{U}\left( 2 \right)=1, \mathbf{C}\left( 1 \right)= 1, \mathbf{CC}\left( 1 \right)=0, \mathbf{CG}\left( 1 \right)=0, \mathbf{A}\left( 19 \right)=0$$

$$\mathbf{U}\left( \mathrm{all} \right)=\frac{10}{19}=0.526, \mathbf{G}\left( \mathrm{all} \right)=\frac{2}{19}=0.105$$

$$\mathbf{GG}\left( \mathrm{all} \right)= 0, \mathbf{UA}\left( \mathrm{all} \right)=\frac{3}{18}=0.167, \mathbf{CC}\left( \mathrm{all} \right)=0, \mathbf{GC}\left( \mathrm{all} \right)= 0, \mathbf{UU}\left( \mathrm{all} \right)=\frac{4}{18}=0.222$$

### *Gibbs free energy changes and enthalpy changes*

Xia et al. (Xia, SantaLucia et al. 1998) calculated ﻿Gibbs free energy and enthalpy changes of all kinds of dinucleotide pairs for INN-HB Nearest-Neighbor Model(Supplementary Table 1.4). Gibbs free energy or enthalpy changes with the number k are the Gibbs free energy or enthalpy changes of the dinucleotide pair at the k and k+1 positions. While Gibbs free energy changes with “all” are a little bit more complicated to calculate using the following equation (Turner and Mathews 2010). The initiation term indicates siRNA-mRNA double strand structure formation, the per AU end is applied once per each AU pair at the end of siRNA, the symmetry term indicates self-complementary duplexes, and the last term is a sum of Gibbs free energy or enthalpy changes over all dinucleotide pairs. The thermodynamic data needed to calculate these equations are shown in the Supplementary Table 1.4.

$$\boldsymbol{\Delta G^{\circ}}\left( k \right)=\boldsymbol{\Delta G^{\circ}}\left( siRNA\left[ k:k+1 \right] \right), k\in[1,19]$$

$$\boldsymbol{\Delta H^{\circ}}\left( k \right)=\boldsymbol{\Delta H^{\circ}}\left( siRNA\left[ k:k+1 \right] \right), k\in[1,19]$$

$$\boldsymbol{\Delta G}_{all}^{^{\circ}}=\boldsymbol{\Delta G}_{initiation}^{^{\circ}}+\boldsymbol{\Delta G}_{per AU end}^{^{\circ}}+\boldsymbol{\Delta G}_{symmetry}^{^{\circ}}+\sum_{k=1}^{18} \boldsymbol{\Delta G^{\circ}}\left( k \right)$$

$$\boldsymbol{\Delta H}_{all}^{^{\circ}}=\boldsymbol{\Delta H}_{initiation}^{^{\circ}}+\boldsymbol{\Delta H}_{per AU end}^{^{\circ}}+\boldsymbol{\Delta H}_{symmetry}^{^{\circ}}+\sum_{k=1}^{18} \boldsymbol{\Delta H^{\circ}}\left( k \right)$$

$${\boldsymbol{\Delta\Delta}\boldsymbol{G}}_{ends}^{^{\circ}}=\boldsymbol{\Delta G^{\circ}}\left( siRNA\left[ 1:2 \right] \right)-\boldsymbol{\Delta G^{\circ}}\left( siRNA\left[ 18:19 \right] \right)+ \boldsymbol{\Delta G}_{per AU end}^{^{\circ}}$$

Take the first siRNA in Huesken dataset “CUAAUAUGUUAAUUGAUUU”, for example,

$$\boldsymbol{\Delta G^{\circ}}\left( 1 \right)=\boldsymbol{\Delta G^{\circ}}\left( siRNA\left[ 1:2 \right] \right)=\boldsymbol{\Delta G^{\circ}}\left( \boldsymbol{CU} \right)=-2.08 kcal/mol$$

$$\boldsymbol{\Delta H^{\circ}}\left( 1 \right)=\boldsymbol{\Delta H^{\circ}}\left( siRNA\left[ 1:2 \right] \right)=\boldsymbol{\Delta H^{\circ}}\left( \boldsymbol{CU} \right)=-10.48 kcal/mol$$

$$\boldsymbol{\Delta G^{\circ}}\left( 2 \right)=\boldsymbol{\Delta G^{\circ}}\left( siRNA\left[ 2:3 \right] \right)=\boldsymbol{\Delta G^{\circ}}\left( \boldsymbol{UA} \right)=-1.33 kcal/mol$$

$$\boldsymbol{\Delta G^{\circ}}\left( 13 \right)=\boldsymbol{\Delta G^{\circ}}\left( siRNA\left[ 13:14 \right] \right)=\boldsymbol{\Delta G^{\circ}}\left( \boldsymbol{UU} \right)=-0.93 kcal/mol$$

$$\boldsymbol{\Delta G^{\circ}}\left( 18 \right)=\boldsymbol{\Delta G^{\circ}}\left( siRNA\left[ 18:19 \right] \right)=\boldsymbol{\Delta G^{\circ}}\left( \boldsymbol{UU} \right)=-0.93 kcal/mol$$

$$\boldsymbol{\Delta H}_{all}^{^{\circ}}=\boldsymbol{\Delta H}_{initiation}^{^{\circ}}+\boldsymbol{\Delta H}_{per AU end}^{^{\circ}}+\boldsymbol{\Delta H}_{symmetry}^{^{\circ}}+\sum_{k=1}^{18} \boldsymbol{\Delta H^{\circ}}\left( k \right)$$

$$=3.61+3.72+0+(-10.48-7.69-6.82-9.38-7.69-9.38-10.44-11.40-6.82-7.69-6.82$$

$$-9.38-6.82-10.44-12.44-9.38-6.82-6.82)$$

$$=-149.38 kcal/mol$$

Therefore, the thermodynamic parameter vector of the example siRNA is :

[-1.6,-2.08,-10.48,0,0,-149.38,0.526,0,0.105,0,0,0,-1.33,0.167,1,1,0,-0.93,0,0,0,-0.93,0.222,0]

Please visit the Github library for more detailed codes.

## RNA-FM embedding

RNA-FM (Zhang, Lang et al. 2024) (RNA Fundation Model) will transform RNA sequences into fixed-length feature vectors for the downstream feature fusion. The siRNA sequences and corresponding truncated mRNA sequences were extracted and formatted into fasta files, serving as input for RNA-FM module. We downloaded pre-trained models of RNA-FM from GoogleDrive (<https://drive.google.com/drive/folders/1VGye74GnNXbUMKx6QYYectZrY7G2pQ_J>), and applied embedding extraction function of RNA-FM to extract feature representations from the siRNA and mRNA sequences. The feature vectors were stored in npy (Numpy) file format, a binary file format used for storing numerical data in Python. The shape of extracted RNA-FM embeddings is $l\times640$, where $l$ is the length of siRNA or mRNA. Therefore, the shape of the siRNA embedding is $19\times640$ and the shape of the mRNA embedding is $57\times640$ for a flanking length of 19 nt. Next, Avg2D pooling was used to reduce the feature dimensions while preserving the most discriminative information. The kernel size of Avg2D pooling was set as $l\times5$, which is the most effective option, so that the 2D feature of $l\times640$ was transformed into the 1D feature of 128. This 1D vector of 128 is the RNA-FM embedding we need for the downstream feature fusion.

## Oligo encoder embedding

The architecture of Oligo encoder consists of a 2D convolutional layer, a max pooling layer, an average pooling layer, a Bidirectional Long Short-Term Memory (BiLSTM) layer, two multi-head transformer encoder layers, and a flatten layer. Oligo encoder takes 19 nt siRNA sequences and 57 nt mRNA sequences $\boldsymbol{S}$ as input. We used one-hot encoding to represent the nucleotide sequences $\boldsymbol{S}$, with each nucleotide (A, U, C, G, X) being assigned a unique binary vector of length 5. As a result, the one-hot embedding $\boldsymbol{E}_{O}$ of one input sequence is a $1\times l\times5$ matrix, where $l$ is the length of siRNA or mRNA.

$\boldsymbol{E}_{O}=OneHot\left( \boldsymbol{S} \right)=[\left[ \boldsymbol{E}_{1},\boldsymbol{E}_{2},\boldsymbol{E}_{3},\cdots,\boldsymbol{E}_{l} \right]],$ $\boldsymbol{E}_{i}\in\mathbb{R}^{5},$ $i=1, 2, 3,\cdots,l$

Next, we used a 2D convolutional layer to further extract features $\boldsymbol{E}_{\boldsymbol{C}}$. The Conv2D layer was configured with an input channel of 1, an output channel of 64, a kernel size of $1\times5$ for siRNA and $5\times5$ for mRNA, a stride of 1, and no padding as described below:

$$\boldsymbol{E}_{\boldsymbol{C}}\boldsymbol{=}Conv2D\left( \boldsymbol{E}_{O} \right)\boldsymbol{=}ReLU\boldsymbol{(}\sum_{i=1}^{64} \boldsymbol{W}_{i}\boldsymbol{\times}\boldsymbol{E}_{O}\boldsymbol{)\in}\mathbb{R}^{64\times l^{'}\times1}$$

$${\boldsymbol{(W}_{i}\boldsymbol{\times}\boldsymbol{E}_{O}\boldsymbol{)}}_{m,n}\boldsymbol{=}\sum_{p=1}^{k_{1}} \sum_{q=1}^{k_{2}} {\boldsymbol{W}_{i,p,q}\boldsymbol{\times}\boldsymbol{E}_{O}}_{m+p-1,n+q-1}$$

, where $l^{'}$ represents 19 for siRNA and 53 for mRNA, ${(k}_{1},k_{2})$ represents the kernel size, ${\boldsymbol{(W}_{i}\times\boldsymbol{E}_{O}\boldsymbol{)}}_{m,n}$ represents the value at position $(m,n)$ in the output feature map, $\boldsymbol{W}_{i,p,q}$ represents the weight at position $(p,q)$ in the $i$-th kernel, and ${\boldsymbol{E}_{O}}_{m+p-1,n+q-1}$ represents the value at position $(m+p-1,n+q-1)$ in the input feature map.

Then, a max pooling layer and an average pooling layer with a sliding window of fixed size $2\times2$ was applied to the transposed $\boldsymbol{E}_{C}$, which reduced the computational burden while retaining the most important information for subsequent layers in the network. The results of pooling layers $\boldsymbol{E}_{A}\boldsymbol{\in}\mathbb{R}^{l^{'}\times32}$ and $\boldsymbol{E}_{C}\boldsymbol{\in}\mathbb{R}^{l^{'}\times32}$ were concatenated with the original one-hot encoded feature $\boldsymbol{E}_{O}\boldsymbol{\in}\mathbb{R}^{l^{'}\times5}$ to form a new feature matrix $\boldsymbol{E}$ $\boldsymbol{\in}\mathbb{R}^{l^{'}\times69}$.

$$\boldsymbol{E}_{A}\boldsymbol{=}AvgPool_{2\times2}\left( \boldsymbol{E}_{C}^{T} \right)\boldsymbol{\in}\mathbb{R}^{l^{'}\times32}$$

$$\boldsymbol{E}_{M}\boldsymbol{=}MaxPool_{2\times2}\left( \boldsymbol{E}_{C}^{T} \right)\boldsymbol{\in}\mathbb{R}^{l^{'}\times32}$$

$$\boldsymbol{E=}Concat\left( \boldsymbol{E}_{O}\boldsymbol{,}\boldsymbol{E}_{A}\boldsymbol{,}\boldsymbol{E}_{M} \right)\boldsymbol{\in}\mathbb{R}^{l^{'}\times69}$$

After the concatenation of $\boldsymbol{E}_{o}$, $\boldsymbol{E}_{A}$ and $\boldsymbol{E}_{M}$, a 2-layer BiLSTM with the input dimension of 69 and the LSTM dimension of 32, was applied to $\boldsymbol{E}$, described as follows:

$$\boldsymbol{E}_{B}\boldsymbol{=}dropout(ReLU\left( BiLSTM\left( \boldsymbol{E} \right) \right)\boldsymbol{)\in}\mathbb{R}^{l^{'}\times64}$$

Multi-head self-attention (MHA) contained 8 heads, and each head was a mapping of query vector $\boldsymbol{Q}$, key vector $\boldsymbol{K}$, value vector $\boldsymbol{V}$, all of which were equal to $\boldsymbol{E}_{B}$. Suppose $N_{h}$ and $d_{k}$ denote the number of heads and the depth of keys, where $N_{h}=8$ and $d_{k}=64$. The output from each head $h$ was computed as follows:

$$\boldsymbol{A}_{h}=dropout(softmax\left( \frac{\boldsymbol{E}_{B}\boldsymbol{W}_{q}\left( \boldsymbol{E}_{B}\boldsymbol{W}_{k} \right)^{T}}{\sqrt{d_{k}}} \right)\boldsymbol{E}_{B}\boldsymbol{W}_{v}\boldsymbol{,} h=1,2,\ldots,N_{h}$$

Then we concatenated the output from each head $h$. The layer normalization (LN), feed-forward network (FFN), and residual connections were utilized to generate the output for each layer. We applied two linear layers with a ReLU activation layer in the FFN layer. Thus, the mechanism is summarized as:

$$\boldsymbol{O}_{0}=[\boldsymbol{A}_{1},\boldsymbol{A}_{2},\ldots\boldsymbol{,A}_{N_{h}}]\boldsymbol{)\in}\mathbb{R}^{l^{'}\times64}$$

$$\boldsymbol{z}_{n}=MHA\left( \left( LN\left( \boldsymbol{O}_{n-1} \right) \right) \right)+\boldsymbol{O}_{n-1}\boldsymbol{\in}\mathbb{R}^{l^{'}\times64}$$

$$\boldsymbol{O}_{n}=FFN\left( \left( LN\left( \boldsymbol{z}_{n} \right) \right) \right)+\boldsymbol{z}_{n}\boldsymbol{\in}\mathbb{R}^{l^{'}\times64}$$

, where$\boldsymbol{O}_{n}$ denotes the output from the $n$-th block and $\boldsymbol{z}_{n}$ denotes the intermediate representation in the $n$-th block.

Finally $\boldsymbol{O}_{n}$ was flattened into a vector $\boldsymbol{O}$ for the downstream feature fusion.

$$\boldsymbol{O}=Flatten(\boldsymbol{O}_{n}\boldsymbol{)}\boldsymbol{\in}\mathbb{R}^{l^{'}*64}$$

## Prediction calculation of OligoFormer

After the calculation module described above, we got thermodynamic embeddings **𝑇**, the RNA-FM embeddings $\boldsymbol{R}_{s}$ and $\boldsymbol{R}_{m}$ for the siRNA and mRNA sequences, and the embeddings $\boldsymbol{O}_{s}$ and $\boldsymbol{O}_{m}$ from the Oligo encoder for the siRNA and mRNA sequences, which was calculated as follows:

$$\boldsymbol{T}=Thermodynamic(\boldsymbol{S}_{s}\boldsymbol{,}\boldsymbol{S}_{\boldsymbol{m}})\boldsymbol{\in}\mathbb{R}^{24}$$

$$\boldsymbol{R}_{s}\boldsymbol{,}\boldsymbol{R}_{m}\boldsymbol{=}RNAFM\boldsymbol{(}\boldsymbol{S}_{s}\boldsymbol{,}\boldsymbol{S}_{\boldsymbol{m}}\boldsymbol{)}\boldsymbol{\in}\mathbb{R}^{128}$$

$$\boldsymbol{O}_{s}\boldsymbol{,}\boldsymbol{O}_{m}\boldsymbol{=}OligoEncoder\boldsymbol{(}\boldsymbol{S}_{s}\boldsymbol{,}\boldsymbol{S}_{\boldsymbol{m}}\boldsymbol{)}\boldsymbol{\in}\mathbb{R}^{l^{'}*64}$$

, where $l^{'}$=19 for siRNA and $l^{'}$=53 for mRNA. The embeddings from these three modules were concatenated together to form a single feature vector $\boldsymbol{X}_{0}$. The prediction calculation of OligoFormer involves a multilayer perceptron (MLP), which takes $\boldsymbol{X}_{0}$ as input and output a scalar value $\hat{Y}$ representing the predicted efficacy of the siRNA sequences.

$$\boldsymbol{X}_{0}= Concat(\boldsymbol{O}_{s}\boldsymbol{,}\boldsymbol{O}_{m},\boldsymbol{R}_{s}\boldsymbol{,}\boldsymbol{R}_{m},\boldsymbol{T})\boldsymbol{\in}\mathbb{R}^{d_{0}}$$

$$\boldsymbol{X}_{1}= dropout(ReLU(\boldsymbol{X}_{0}\boldsymbol{W}_{1}+\boldsymbol{B}_{1}))\boldsymbol{\in}\mathbb{R}^{d_{1}}$$

$$\boldsymbol{X}_{2}= dropout(ReLU(\boldsymbol{X}_{1}\boldsymbol{W}_{2}+\boldsymbol{B}_{2}))\boldsymbol{\in}\mathbb{R}^{d_{2}}$$

$$\hat{Y}=softmax(\boldsymbol{X}_{2}\boldsymbol{W}_{3}+\boldsymbol{B}_{3})\boldsymbol{\in}\mathbb{R}^{d_{3}}$$

, where $\boldsymbol{W}_{1}\boldsymbol{\in}\mathbb{R}^{d_{0}\times d_{1}}$, $\boldsymbol{W}_{2}\boldsymbol{\in}\mathbb{R}^{d_{1}\times d_{2}}$, $\boldsymbol{W}_{3}\boldsymbol{\in}\mathbb{R}^{d_{2}\times d_{3}}$ are the weight matrices as parameters; $d_{0}=$ 4888 is the dimension of $\boldsymbol{X}_{0}$ as MLP input; $d_{1}=$ 256, $d_{2}=$ 64, $d_{3}=$ 2 are numbers of neurons for three fully connected neural network layers; $\boldsymbol{B}_{1}\boldsymbol{\in}\mathbb{R}^{d_{1}}$, $\boldsymbol{B}_{2}\boldsymbol{\in}\mathbb{R}^{d_{2}}$, $\boldsymbol{B}_{3}\boldsymbol{\in}\mathbb{R}^{d_{3}}$ are the terms of bias; ReLU is an activation function; softmax is a normalized exponential function; dropout is a dropout neural network layer with certain probability; $\hat{Y}$ is the predicted siRNA efficacy.

## Details of model training and test

### *Parameters of Model training*

In the OligoFormer model, we utilized the PyTorch package in Python v3.7.16 to implement the network architecture. For model training and test, mean squared error (MSE) was chosen as the loss function, which quantified the average squared difference between predicted and actual efficacy values, penalizing larger errors more severely than smaller ones. We implemented Adam optimizer and weight decay function in the PyTorch package for optimized learning rate scheduling, which dynamically adjusted the learning rate during training and enhanced model convergence and performance. During model training, we optimized hyperparameters such as learning rate, weight decay, batch size, dropout probability, and epoch numbers inside the training set only. The learning rate was set to 0.0001, weight decay was set to 0.999 to ensure convergence, batch size was set to 16, the number of training epochs was set to 200 and early stopping was set to 30 epochs to prevent overfitting. Additionally, a fixed seed value of 42 was used to ensure reproducibility of results.

### *Evaluation metrics*

Four evaluation metrics, area under the receiver operating characteristic curve (AUC), F1 score, area under the precision-recall curve (AUPRC), and Pearson correlation coefficient (PCC) were used to evaluate the prediction performance. By employing these training parameters and validation strategies, the OligoFormer model was trained effectively to accurately predict the efficacy of siRNA sequences. The following shows the calculation of these metrics for evaluation.

$$MSE \left( Mean Square Error \right)=\frac{\sum_{i=1}^{n} \left( \hat{y}-y \right)^{2}}{n}$$

$$PCC=\frac{E\left( y\hat{y} \right)-E\left( y \right)E(\hat{y})}{\sqrt{E\left( y^{2} \right)-E^{2}(y)}\sqrt{E\left( \hat{y}^{2} \right)-E^{2}(\hat{y})}}=\frac{\sum_{i=1}^{n} y\hat{y}-\frac{\sum_{i=1}^{n} y\sum_{i=1}^{n} \hat{y}}{n}}{\sqrt{(\sum_{i=1}^{n} y^{2}-\frac{\left( \sum_{i=1}^{n} y \right)^{2}}{n})(\sum_{i=1}^{n} \hat{y}^{2}-\frac{\left( \sum_{i=1}^{n} \hat{y} \right)^{2}}{n})}}$$

$$TP \left( True Positive \right)=The number of correctly predicted positives$$

$$TN \left( True Negative \right)=The number of correctly predicted negatives$$

$$FP \left( False Positive \right)=The number of incorrectly predicted positives$$

$$FN \left( False Negative \right)=The number of incorrectly predicted negatives$$

$$Precision=\frac{TP}{TP+FP}$$

$$Sensitivity=Recall=\frac{TP}{TP+FN}$$

$$Specificity=\frac{TN}{TN+FP}$$

$$FDR=\frac{FP}{FP+TN}$$

$$Accuracy=\frac{TP+TN}{TP+FP+TN+FN}$$

$$F1 score=2\times\frac{Precision\times Recall}{Precision+Recall}$$

### *Cross validation*

### During model test, we employed a 5-fold cross-validation strategy on the Huesken, Mixset, and Takayuki datasets for intra-dataset validation and ﻿we trained models on the Huesken dataset and evaluated the performance on the Mixset dataset for inter-dataset validation. We also performed cross cell line validation to ensure that our model could maintain high performance when applied to different cell lines, after excluding similar sequences. The Huesken dataset mainly included the H1299 cell line, with some other cell lines that did not coincide with cell lines of the other datasets and were called as the merged H1299 cell line (mH1299). Therefore, we focused on 5 different cell lines: mH1299, HaCaT, HEK293, HeLa, and T24. For each pair of cell lines, we trained the model on mH1299 dataset and tested it on another cell line dataset, ensuring the performance of OligoFormer was evaluated on unseen data from a different cell line. The results (Supplementary Table 1.5) demonstrate that OligoFormer maintains robust performance across different cell lines.

## Comparison methods

### For benchmarking, we compared six representative models for siRNA design, i.e., Monopoli-RF (Monopoli, Korkin et al. 2023), OligoWalk (Lu and Mathews 2008), siRNAPred (Kumar 2009), i-Score (Ichihara, Murakumo et al. 2007), s-Biopredsi (Huesken, Lange et al. 2005), and DSIR (Vert, Foveau et al. 2006). This section introduces how these comparison methods were implemented and the prediction results of test dataset are listed (Supplementary Table 3).

### *Monopoli-RF*

### Monopoli et al (Monopoli, Korkin et al. 2023). from Khvorova lab has recently published a machine learning approach to predict siRNA efficacy. This approach used asymmetric trichotomous grouping method with two independently selected thresholds: one threshold defined effective siRNAs and the other defined ineffective siRNAs, which split siRNA datsets into “effeactive”, “undefined”, and “ineffective” these three categories. Then it built a series of random forest (RF) models with k-fold cross-validation to form a final model to predict effective and ineffective siRNAs. The code for this model is available on Github (<https://github.com/kmonopoli/trichotomous_data_partitioning_model_building>). Since the author did not name the model, we called it Monopoli-RF for short. In order to maintain the consistency of the other comparison methods, the threshold for defining effective or ineffective siRNA was set at 70% inhibition efficiency, thus there were no undefined siRNA partitions. We also trained the model on the original training data from the author's previous paper (Shmushkovich, Monopoli et al. 2018) and tested the performance (Supplementary Table 1.5).

### *OligoWalk*

### OligoWalk employs the nearest-neighbor thermodynamic model to calculate the binding free energy (∆G) of RNA duplex formation between an siRNA and the target mRNA. OligoWalk can predict the efficacy of siRNAs based on thermodynamic principles and molecular interactions. Because the OligoWalk software is embedded in the RNAstructure software, we downloaded RNAStructure (<https://rna.urmc.rochester.edu/RNAstructure.html>) and implemented OligoWalk model locally on the Linux platform. OligoWalk takes the fatsa file of the mRNA as input, and outputs efficacy predictions of all corresponding siRNAs. To reduce the calculation amount, we customed the start and end positions of mRNA by setting parameters --start and--end to the maximum range of siRNAs to be calculated. We set the length of predicted siRNA to 19, and obtained the efficacy predictions of siRNAs.

### *siRNAPred*

### siRNAPred involves support vector machines (SVMs) to predict efficacy of both 21mer and 19mer siRNAs. Nucleotide frequency features i.e. occurrence of mono to penta nucleotide string in a siRNA sequence were in develop hybrid-4 SVM method, binary patterns i.e. presence or absence of A, G, C, T/U nucleotides at each position in a siRNA sequence were used in binary pattern SVM method, and both methods were combined in hybrid-7 SVM method. We used the siRNAPred web server (https://webs.iiitd.edu.in/raghava/sirnapred/) to implement siRNAPred model. We chose efficacy prediction for 19mer and set the method as hybrid-7 method to get the efficacy predictions of siRNAs.

### *i-Score, s-Biopredsi, and DSIR*

### i-Score (inhibitory-Score) utilized a linear regression model on 2431 siRNAs to predict active siRNAs. It exclusively comprised of nucleotide preferences at each position and no other parameters. For s-Biopredsi, each siRNA sequence was input into the input layer neural network while the reporter data were utilized to update the weights between the network nodes with a learning rate of 0.1. This process was repeated ten times for each siRNA sequence and its corresponding target inhibition value. The final output was obtained by averaging the signals of the respective output nodes from all five networks. DSIR (Design of SIRna) is a simple linear model combining basic features of siRNA sequences for siRNA efficacy prediction. The i-Score Designer calculates nine different siRNA designing scores including i-Score, s-Biopredsi, and DSIR. And it provides an accessible web server (https://www.med.nagoya-u.ac.jp/neurogenetics/i_Score/i_score.html) where we can input mRNA and calculate efficacy predictions of 19 nt siRNA traversing the full length of mRNA online.

## Ablation study

Ablation study was conducted to evaluate the impact of different features and modules on the performance of OligoFormer. It was divided into two parts: ablation study of different features, and ablation study of different modules (Supplementary Table 4).

### *Ablation study of different features*

We examined the performance of different combinations of 4 types of features: siRNA sequences, mRNA sequences, RNA-FM embeddings, and thermodynamic parameters (TD). 15 feature sets were generated by combining these feature types: siRNA only, mRNA only, RNA-FM only, TD only, siRNA + mRNA, siRNA + RNA-FM, siRNA + TD, mRNA + RNA-FM, mRNA + TD, RNA-FM + TD, siRNA + mRNA + RNA-FM, siRNA + mRNA + TD, siRNA + RNA-FM + TD, mRNA + RNA-FM + TD, and combination of all 4 features. The results indicated that the full combination of all 4 features provided the best performance. The inclusion of RNA-FM features significantly enhanced the model's understanding of RNA sequences, while thermodynamic parameters contributed to the interpretation of molecular stability and interactions. A notable decline in performance was observed when features particularly RNA-FM and siRNA features were removed, which means these two features are crucial for capturing the complexities of RNA interactions. The thermodynamic parameters and mRNA features showed a less important impact when excluded, indicating that while they are beneficial, the model can still maintain a reasonable level of performance without them.

### *Ablation study of different modules*

We focused on three basic modules of the OligoFormer architecture: Encoder layer, Conv2D, and BiLSTM. By creating models with different combinations of these modules, we evaluated the contribution of each to the overall performance, resulting in 7 module sets. Encoder layer Only, Conv2D only, BiLSTM only, Encoder layer + Conv2D, Encoder layer + BiLSTM, Conv2D + BiLSTM, and combination of all 3 modules. The full model achieved the best performance, demonstrating the complementary strengths of these modules. The Encoder layer as the core transformer encoder can capture the initial sequence representations, Conv2D can identify local patterns, and BiLSTM are crucial for capturing dependencies within the sequences. Removing any single component resulted in a drop in performance. Specifically, the absence of the Encoder layer led to the most significant decline, showing its critical role in initial sequence processing. The removal of Conv2D also significantly impacted performance. The impact of the exclusion of BiLSTM was slightly less severe compared to the other two modules.

## Off-target

### *Functionality evaluation*

To ensure the activity of the siRNA, some sequences with features known to be detrimental to RNAi functionality need to be removed (Birmingham, Anderson et al. 2007). (1) GC contents. High GC content may inhibit the dissociation of the duplex which is essential for RISC loading while very low GC content may cause lower target affinity and specificity. A range of 30–65% GC is considered optimal for identifying effective siRNAs and is generally utilized among potency-based siRNA design algorithms (Boudreau, Spengler et al. 2013). (2) five or more of any single bases in a row. Repeated bases have been shown to reduce functionality and selectivity and A or U stretches may additionally target regulatory motifs. (3) six or more consecutive G’s and/or C’s. Such regions have pronounced local stability which greatly inhibits duplex dissociation. (4) palindromic sequence. Internal complementary stretches more than three bases long may reduce the effective concentration of the mature duplex. Unstructured siRNAs result in higher efficacy. All of the above criteria are filtered by default.

### *Toxicity evaluation*

Identification of putative toxic inducible factors within siRNAs provides a basis for the rational design of synthetic siRNAs that might avoid activating the innate immune response and improve the likelihood of passing toxicology tests. The toxicity of sequences are assessed and screened from two primary perspectives: (1) immunostimulatory motif. Toll-like receptors (TLRs) protect the host from pathogens by detecting infectious external agents including double-stranded RNA. Therefore, siRNAs may be recognized by the immune system as an unwanted invader and trigger an immune response (Jackson and Linsley 2010). Some known immunostimulatory motifs (GUCCUUCAA, UGUGU and CUGAAUU) will be filtered by default (Fakhr, Zare et al. 2016). (2) seed toxicity. Previous study has confirmed a cell type- and species- independent form of toxicity evoked by the 6mer seed sequence in siRNAs(Gao, Putzbach et al. 2018). All 4,096 6mer seed sequences and their corresponding cell viability have been downloaded (<https://www.6merdb.org/>). By default, siRNA 6mer seed regions with a cell viability of less than 50% are removed. Users can also customize the filter threshold ranging from 0 to 100%.

### *Incorporation of off-target search*

PITA and TargetScan Context++ scores are utilized to evaluate the siRNA off-target effects. Both methods are capable of accepting 3'UTR sequences, siRNA sequences, and ORF sequences as inputs. UTR and ORF sequences are downloaded from TargetScan (<https://www.targetscan.org/cgi-bin/targetscan/data_download.vert80.cgi>) . Note that the ORF sequences and the UTR sequences need to correspond with each other. Keep the default settings for other parameters.

If there is a possibility of siRNA and mRNA binding, the program will output a predicted off-target score (miRNA-like off-target score), otherwise it will not output a result, which means that this siRNA may not be off-target on this mRNA. For PITA, usually sequences with an off-target score below -10 have a greater chance of missing the target. For TargetsScan, there is no fixed threshold for determining whether the siRNA is off-target. This requires the users to choose the appropriate threshold based on practical needs and biological understanding. By default we set the threshold to 1. Users can also change the size of the two thresholds. Sequences that are predicted to miss the target by either method are filtered.

To test the off-target search module, off-target signatures of six different siRNAs targeting *APOB* gene in two human cell lines, HUH7 and PLC/PRF/5, were collected and defined as Burchard dataset (Burchard, Jackson et al. 2009). RNA was extracted at 6, 12, 24 and 48 hours upon transfection and gene expression was measured with microarray. We obtained the expression profile from the Gene Expression Omnibus database with accession number GSE14073 and performed the differential expression analysis through GEO2R interface. For each cell line and each siRNA, 6h and 12h samples were treated as technical replicates and log_2_ fold changes (log_2_FC) of transcripts between mock and siRNA transfection were gained. Transcripts with log_2_FC below -0.4 are treated as off-target sequences and labeled as 1 while transcripts with log_2_FC between -0.4 and 0 were treated as off-target sequences and labeled as 0. We fed the sequences of these transcripts into the off-target prediction module and output the predictions with the default settings. Accuracy, sensitivity, specificity and F1-score of off-target prediction module were calculated.

## Mismatch Handling Module

We provide an optional module to handle mismatches to enhance OligoFormer’s utility for designing siRNAs with differential activity on pathogenic alleles. This module is based on experimental data from this study (Becker, Ober-Reynolds et al. 2019), in which the dataset includes hsa-miR-21 and has-let-7a with specific mismatches and their corresponding association rate $k_{on}$. And the ratio of $k_{on}$ of a mismatched sequence to that of the perfectly matched sequence serves as the label for our model. Each nucleotide sequence was one-hot encoded and converted into numerical matrices suitable for input of a neural network. A convolutional neural network (CNN) was employed to learn the impact of mismatches on binding efficiency, which took an original RNA and a mismatched RNA as input and output the $k_{on}$ ratio. More details of model training and test of mismatch module are on our Github repository. The final model was integrated into OligoFormer as an optional module. Users can select “-m” option and input a perfectly matched siRNA sequence along with a mismatched sequence of equal length. The module processes these inputs through the CNN and outputs the predicted $k_{on}$ ratio, indicating the binding efficiency of the mismatched sequence relative to the perfectly matched one.

## User-friendly OligoFormer based on Docker

In order to create the docker image for OligoFormer, we made a customed dockerfile. We specified the base image as slim Python 3.8 and set the working directory to '/app' in the dockerfile, where OligoFormer and its dependencies will be installed. We then copied the OligoFormer source code and any required configuration files into the '/app' directory within the image. Next, we used the 'pip' package manager to install the necessary Python packages listed in OligoFormer's requirements.txt file. Additionally, we also installed some supplementary tools and dependencies required by OligoFormer to ensure its functionality within the docker container. Finally, we built the docker image using the 'docker build' command to complie the dockerfile instructions into a docker image encapsulating OligoFormer and its dependencies. We then uploaded the docker image of OligoFormer to the docker hub as a publicly accessible image.

With just two simple commands of pulling the OligoFormer docker image and running the OligoFormer container, users can quickly set up and utilize OligoFormer within a docker container environment. Firstly, fetch the latest OligoFormer docker image from the official docker hub repository by the command of “docker pull oligoformer:v1.0”. Secondly, create and run the docker container by the command of “docker run -it --name oligoformer-container -dt --restart unless-stopped oligoformer:v1.0 && docker exec -it oligoformer-container bash”. In this way, users can utilize OligoFormer conveniently on docker container environment in their workflows for oligonucleotide design.

# 2 Supplementary Figures

**
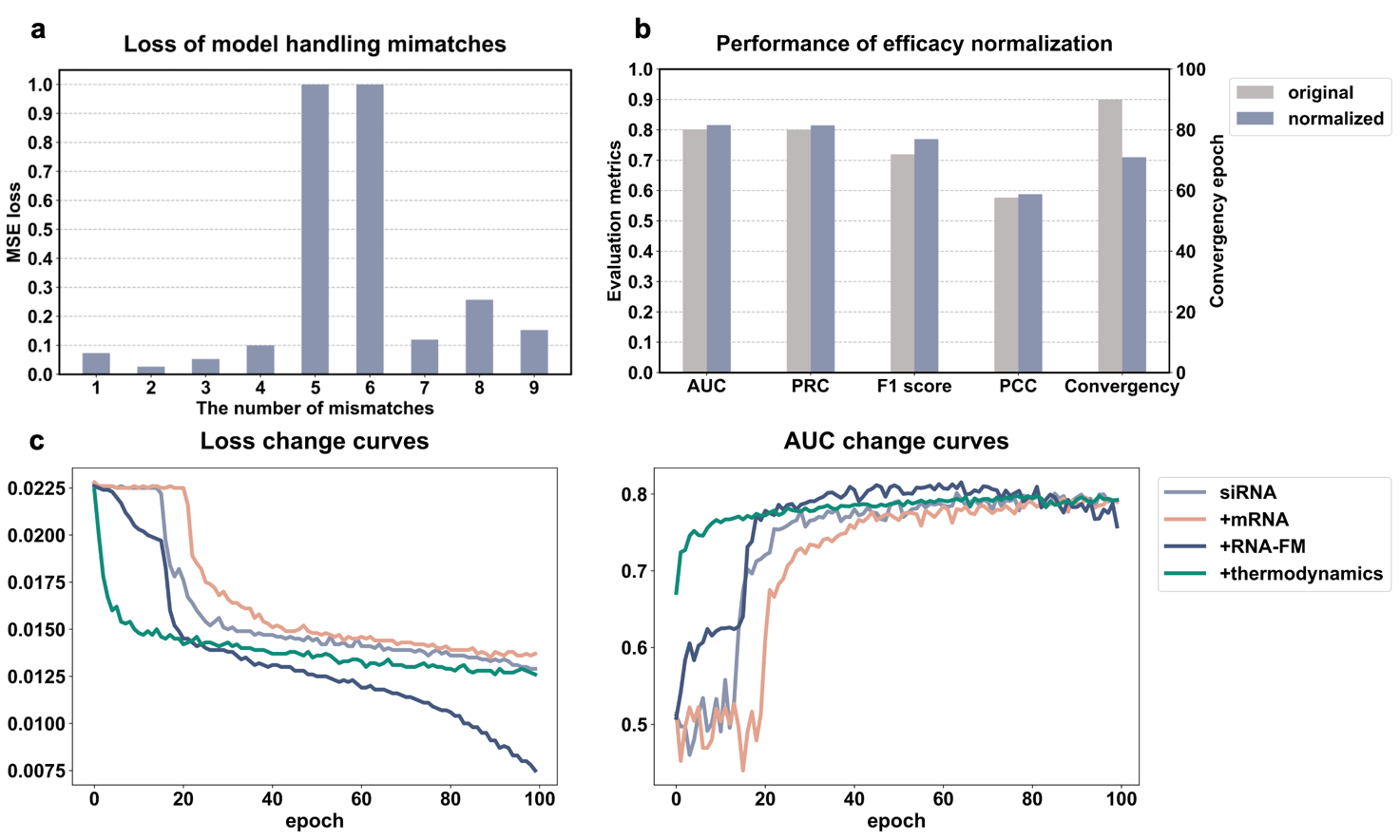
**

## Supplementary Fig. 1: Analysis of model loss and convergence speed.

(a) Comparison of performance between original efficacy and normalized efficacy. (b) MSE loss of model for handling siRNA with some mismatches. (c) Loss change curves of models with different features. (d) AUC change curves of models with different features. The addition of mRNA features will slow down the convergence speed of the model, and the addition of RNA-FM and thermodynamic features will greatly accelerate the convergence of the model.

**
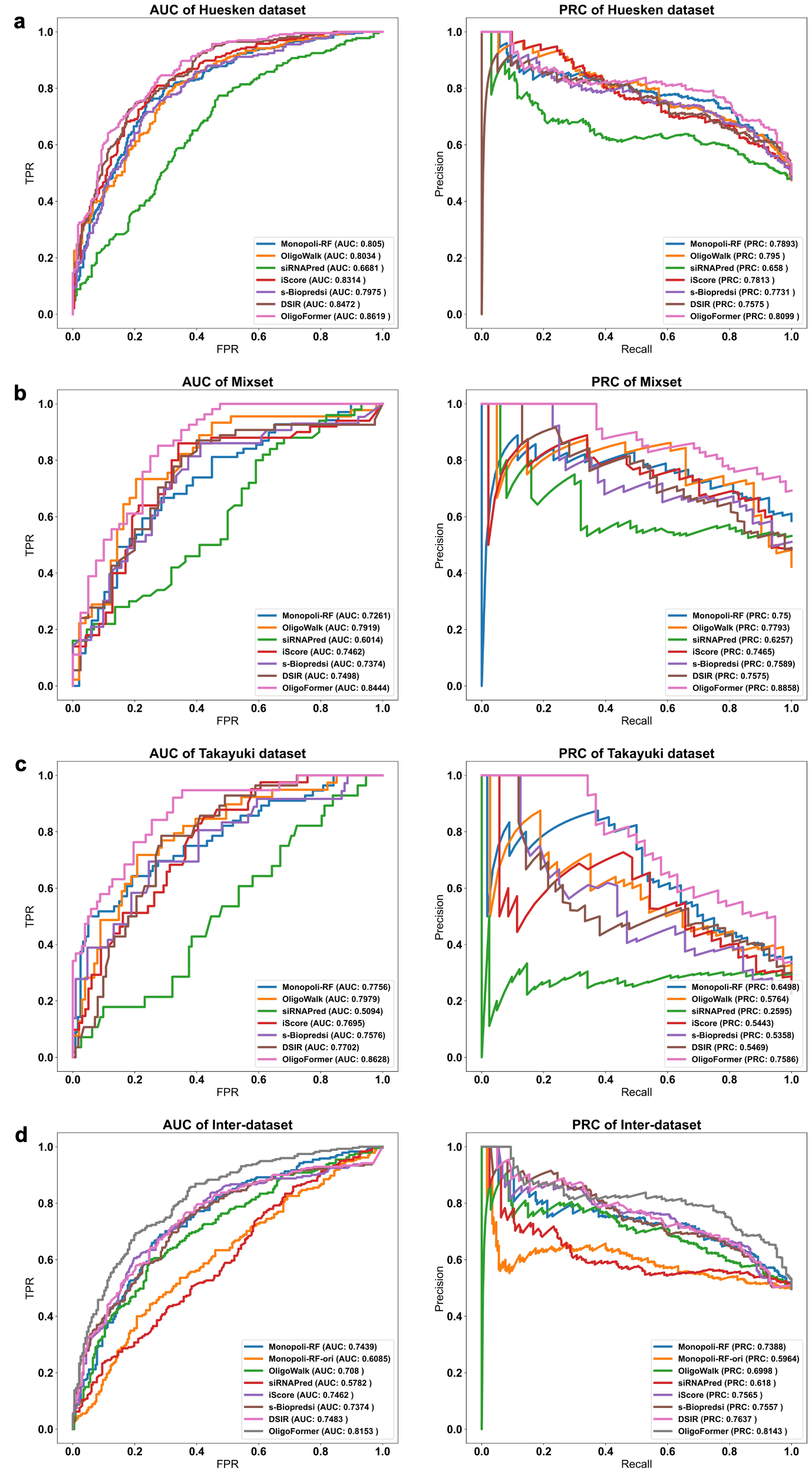
**

## Supplementary Fig. 2: AUC and PRC of different methods.

AUC and PRC of Monopoli-RF, OligoWalk, siRNAPred, iScore, s-Biopredsi, DSIR, and OligoFormer, where (a) Huesken dataset, (b) Mixset, and (c) Takayuki dataset are intra-dataset validation, and (d) is inter-dataset validation.

**
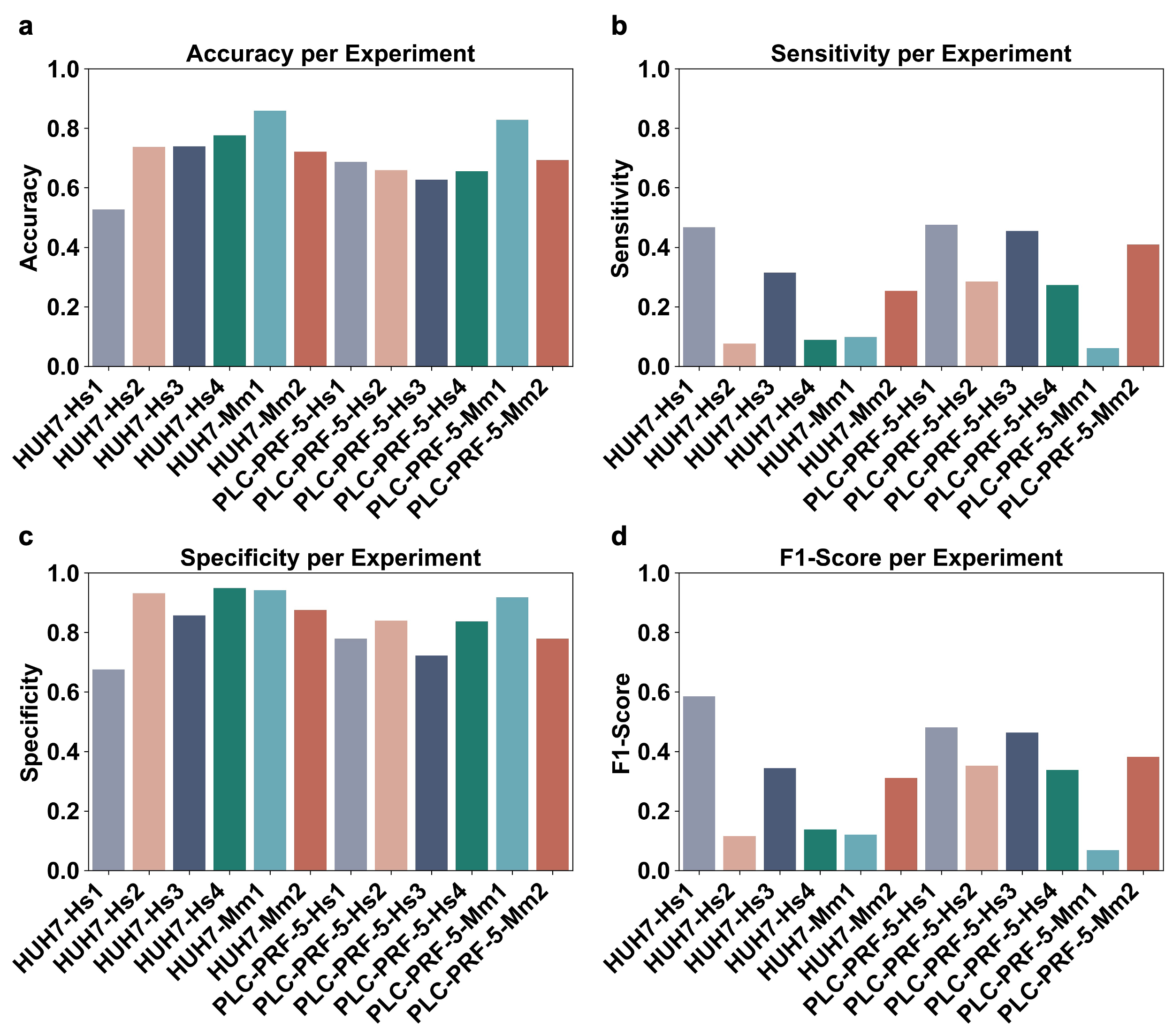
**

## Supplementary Fig. 3: Performance of off-target prediction module.

Accuracy (a), sensitivity (b), specificity (c), and F1-score (d) of off-target prediction module in Burchard dataset. For each of the six siRNAs from the Burchard dataset, we ran off-target prediction module for HUH7 and PLC/PRF/5 cell lines.

**
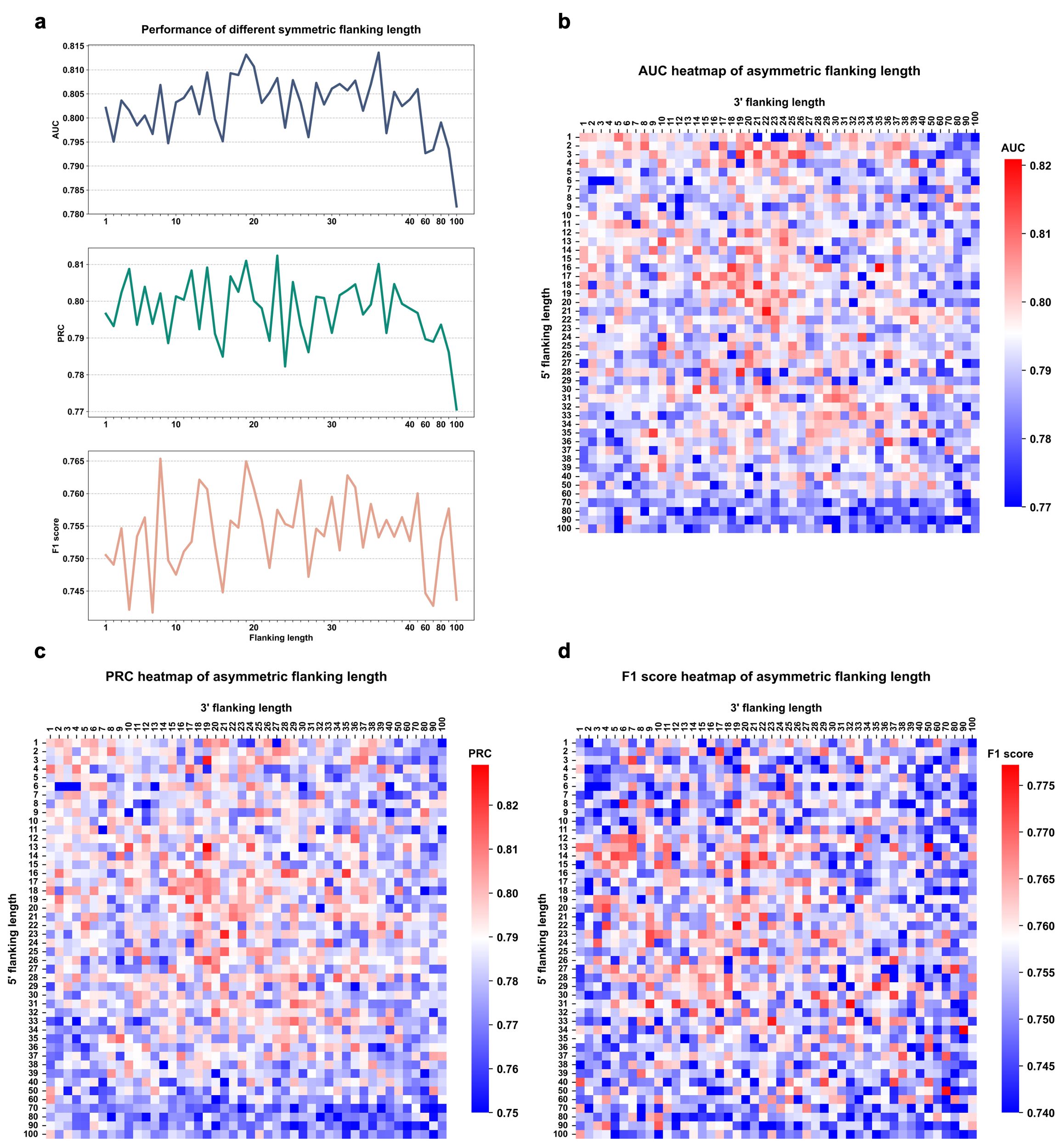
**

## Supplementary Fig. 4: Performance of symmetric and asymmetric flanking lengths.

(a) AUC, PRC, and F1 score for symmetric flanking lengths range from 1 nt to 100 nt for inter-dataset validation. AUC (b), PRC (c), and F1 score (d) heatmap for asymmetric flanking lengths range from 1 nt to 100 nt for inter-dataset validation.

**
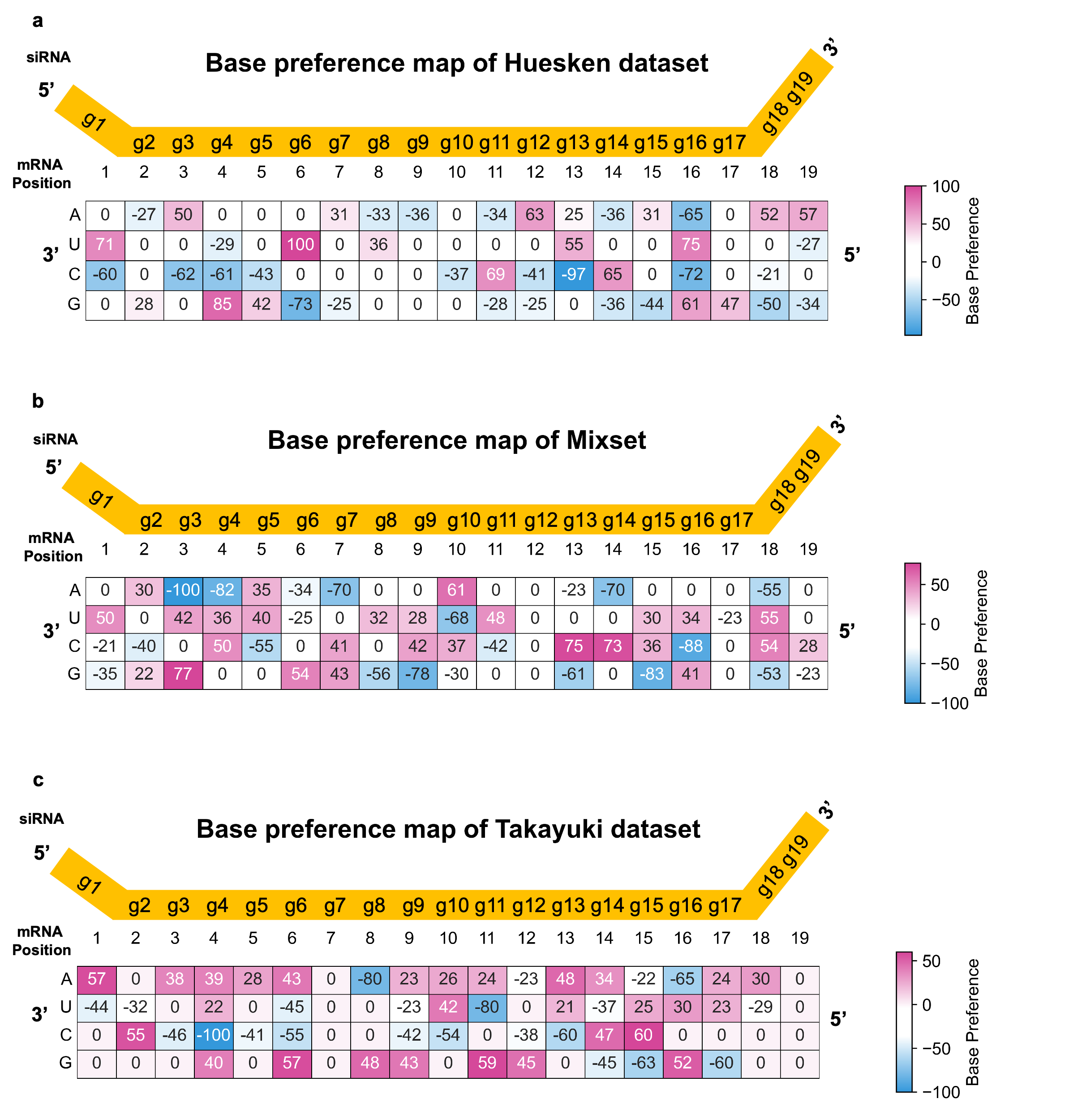
**

## Supplementary Fig. 5: Base preference maps of different datasets.

Base preference maps for Huesken dataset (a), Mixset (b), and Takayuki dataset (c). siRNA is from 5 'to 3', mRNA is from 3 'to 5'. A larger absolute value of weight indicates greater importance for prediction, while a weight of zero indicates no importance of that base at that position.

## Supplementary Fig. 6: Generation and processing of siRNA efficacy.

(a) Illustration of generation of siRNA efficacy, taking one target mRNA as an example. (b) Illustration of efficacy normalization during training and test, and inverse normalization during inference.

# References

Becker, W. R., B. Ober-Reynolds, K. Jouravleva, S. M. Jolly, P. D. Zamore and W. J. Greenleaf (2019). "High-Throughput Analysis Reveals Rules for Target RNA Binding and Cleavage by AGO2." Mol Cell **75**(4): 741-755 e711.

Birmingham, A., E. Anderson, K. Sullivan, A. Reynolds, Q. Boese, D. Leake, J. Karpilow and A. Khvorova (2007). "A protocol for designing siRNAs with high functionality and specificity." Nat Protoc **2**(9): 2068-2078.

Boudreau, R. L., R. M. Spengler, R. H. Hylock, B. J. Kusenda, H. A. Davis, D. A. Eichmann and B. L. Davidson (2013). "siSPOTR: a tool for designing highly specific and potent siRNAs for human and mouse." Nucleic Acids Res **41**(1): e9.

Burchard, J., A. L. Jackson, V. Malkov, R. H. Needham, Y. Tan, S. R. Bartz, H. Dai, A. B. Sachs and P. S. Linsley (2009). "MicroRNA-like off-target transcript regulation by siRNAs is species specific." RNA **15**(2): 308-315.

Fakhr, E., F. Zare and L. Teimoori-Toolabi (2016). "Precise and efficient siRNA design: a key point in competent gene silencing." Cancer Gene Ther **23**(4): 73-82.

Gao, Q. Q., W. E. Putzbach, A. E. Murmann, S. Chen, A. A. Sarshad, J. M. Peter, E. T. Bartom, M. Hafner and M. E. Peter (2018). "6mer seed toxicity in tumor suppressive microRNAs." Nat Commun **9**(1): 4504.

Huesken, D., J. Lange, C. Mickanin, J. Weiler, F. Asselbergs, J. Warner, B. Meloon, S. Engel, A. Rosenberg, D. Cohen, M. Labow, M. Reinhardt, F. Natt and J. Hall (2005). "Design of a genome-wide siRNA library using an artificial neural network." Nat Biotechnol **23**(8): 995-1001.

Ichihara, M., Y. Murakumo, A. Masuda, T. Matsuura, N. Asai, M. Jijiwa, M. Ishida, J. Shinmi, H. Yatsuya, S. Qiao, M. Takahashi and K. Ohno (2007). "Thermodynamic instability of siRNA duplex is a prerequisite for dependable prediction of siRNA activities." Nucleic Acids Research **35**(18).

Jackson, A. L. and P. S. Linsley (2010). "Recognizing and avoiding siRNA off-target effects for target identification and therapeutic application." Nat Rev Drug Discov **9**(1): 57-67.

Kumar, M., S. Lata, and G. P. S. Raghava (2009). "siRNApred: SVM based method for predicting efficacy value of siRNA." CSIR-IMTECH.

Ladunga, I. (2007). "More complete gene silencing by fewer siRNAs: transparent optimized design and biophysical signature." Nucleic Acids Res **35**(2): 433-440.

Lu, Z. J. and D. H. Mathews (2008). "OligoWalk: an online siRNA design tool utilizing hybridization thermodynamics." Nucleic Acids Res **36**(Web Server issue): W104-108.

Monopoli, K. R., D. Korkin and A. Khvorova (2023). "Asymmetric trichotomous partitioning overcomes dataset limitations in building machine learning models for predicting siRNA efficacy." Mol Ther Nucleic Acids **33**: 93-109.

Needleman, S. B. and C. D. Wunsch (1970). "A general method applicable to the search for similarities in the amino acid sequence of two proteins." J Mol Biol **48**(3): 443-453.

Shmushkovich, T., K. R. Monopoli, D. Homsy, D. Leyfer, M. Betancur-Boissel, A. Khvorova and A. D. Wolfson (2018). "Functional features defining the efficacy of cholesterol-conjugated, self-deliverable, chemically modified siRNAs." Nucleic Acids Res **46**(20): 10905-10916.

Turner, D. H. and D. H. Mathews (2010). "NNDB: the nearest neighbor parameter database for predicting stability of nucleic acid secondary structure." Nucleic Acids Res **38**(Database issue): D280-282.

Vert, J. P., N. Foveau, C. Lajaunie and Y. Vandenbrouck (2006). "An accurate and interpretable model for siRNA efficacy prediction." BMC Bioinformatics **7**: 520.

Xia, T., J. SantaLucia, Jr., M. E. Burkard, R. Kierzek, S. J. Schroeder, X. Jiao, C. Cox and D. H. Turner (1998). "Thermodynamic parameters for an expanded nearest-neighbor model for formation of RNA duplexes with Watson-Crick base pairs." Biochemistry **37**(42): 14719-14735.

Zhang, Y., M. Lang, J. Jiang, Z. Gao, F. Xu, T. Litfin, K. Chen, J. Singh, X. Huang, G. Song, Y. Tian, J. Zhan, J. Chen and Y. Zhou (2024). "Multiple sequence alignment-based RNA language model and its application to structural inference." Nucleic Acids Res **52**(1): e3.
